# Epigenetic–smoking interaction reveals histologically heterogeneous effects of *TRIM27* DNA methylation on overall survival among early‐stage NSCLC patients

**DOI:** 10.1002/1878-0261.12785

**Published:** 2020-09-03

**Authors:** Xinyu Ji, Lijuan Lin, Sipeng Shen, Xuesi Dong, Chao Chen, Yi Li, Ying Zhu, Hui Huang, Jiajin Chen, Xin Chen, Liangmin Wei, Jieyu He, Weiwei Duan, Li Su, Yue Jiang, Juanjuan Fan, Jinxing Guan, Dongfang You, Andrea Shafer, Maria Moksnes Bjaanæs, Anna Karlsson, Maria Planck, Johan Staaf, Åslaug Helland, Manel Esteller, Yongyue Wei, Ruyang Zhang, Feng Chen, David C. Christiani

**Affiliations:** ^1^ Department of Biostatistics Center for Global Health School of Public Health Nanjing Medical University Nanjing China; ^2^ Department of Environmental Health Harvard T.H. Chan School of Public Health Boston MA USA; ^3^ China International Cooperation Center for Environment and Human Health Nanjing Medical University Nanjing China; ^4^ Department of Epidemiology and Biostatistics School of Public Health Southeast University Nanjing China; ^5^ Department of Biostatistics University of Michigan Ann Arbor MI USA; ^6^ Department of Bioinformatics School of Biomedical Engineering and Informatics Nanjing Medical University Nanjing China; ^7^ Pulmonary and Critical Care Division Department of Medicine Massachusetts General Hospital and Harvard Medical School Boston MA USA; ^8^ Department of Cancer Genetics Institute for Cancer Research Oslo University Hospital Oslo Norway; ^9^ Division of Oncology and Pathology Department of Clinical Sciences Lund and CREATE Health Strategic Center for Translational Cancer Research Lund University Lund Sweden; ^10^ Institute of Clinical Medicine University of Oslo Oslo Norway; ^11^ Josep Carreras Leukaemia Research Institute Badalona, Barcelona Spain; ^12^ Centro de Investigacion Biomedica en Red Cancer Madrid Spain; ^13^ Institucio Catalana de Recerca i Estudis Avançats Barcelona Spain; ^14^ Physiological Sciences Department School of Medicine and Health Sciences University of Barcelona Barcelona Spain; ^15^ State Key Laboratory of Reproductive Medicine Nanjing Medical University Nanjing China; ^16^ Jiangsu Key Lab of Cancer Biomarkers, Prevention and Treatment Cancer Center Collaborative Innovation Center for Cancer Personalized Medicine Nanjing Medical University Nanjing China

**Keywords:** DNA methylation, interaction, non‐small‐cell lung cancer, overall survival, prognosis, *TRIM27*

## Abstract

Tripartite motif containing 27 (*TRIM27*) is highly expressed in lung cancer, including non‐small‐cell lung cancer (NSCLC). Here, we profiled DNA methylation of lung adenocarcinoma (LUAD) and lung squamous cell carcinoma (LUSC) tumours from 613 early‐stage NSCLC patients and evaluated associations between CpG methylation of *TRIM27* and overall survival. Significant CpG probes were confirmed in 617 samples from The Cancer Genome Atlas. The methylation of the CpG probe cg05293407*_TRIM27_* was significantly associated with overall survival in patients with LUSC (HR = 1.65, 95% CI: 1.30–2.09, *P* = 4.52 × 10^−5^), but not in patients with LUAD (HR = 1.08, 95% CI: 0.87–1.33, *P* = 0.493). As incidence of LUSC is associated with higher smoking intensity compared to LUAD, we investigated whether smoking intensity impacted on the prognostic effect of cg05293407*_TRIM27_* methylation in NSCLC. LUSC patients had a higher average pack‐year of smoking (37.49_LUAD_ vs 54.79_LUSC_, *P* = 1.03 × 10^−19^) and included a higher proportion of current smokers than LUAD patients (28.24%_LUAD_ vs 34.09%_LUSC_, *P* = 0.037). cg05293407*_TRIM27_* was significantly associated with overall survival only in NSCLC patients with medium–high pack‐year of smoking (HR = 1.58, 95% CI: 1.26–1.96, *P* = 5.25 × 10^−5^). We conclude that cg05293407*_TRIM27_* methylation is a potential predictor of LUSC prognosis, and smoking intensity may impact on its prognostic value across the various types of NSCLC.

## Graphical abstract

Among early‐stage non‐small‐cell lung cancer (NSCLC) patients, cg05293407*_TRIM27_* was significantly and exclusively associated with survival of lung squamous cell carcinoma patients, who had higher smoking intensity compared to lung adenocarcinoma patients. Generally, the significant association between cg05293407*_TRIM27_* and survival only remained in NSCLC patients having medium‐to‐high pack‐year of smoking. The cg05293407*_TRIM27_*‐smoking synergistic interaction might account for histologically heterogeneous effects of *TRIM27* DNA methylation on NSCLC survival.

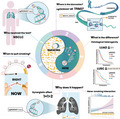

**Abbreviations**
CI: confidence interval
FDR: false discovery rate
HR: hazard ratio
LUAD: lung adenocarcinomas
LUSC: lung squamous cell carcinomas
NSCLC: non‐small‐cell lung cancer
OS: overall survival
QC: quality control
SD: standard deviation
TCGA: The Cancer Genome Atlas
*TRIM27*: tripartite motif containing 27
TSS: transcription start site

## 1 Introduction

Lung cancer is the most commonly diagnosed cancer, accounting for 11.6% of total cases in 2018 [1]. More than 85% of lung cancer cases are non‐small‐cell lung cancer (NSCLC), of which lung adenocarcinoma (LUAD) and lung squamous cell carcinoma (LUSC) are the most common subtypes [2, 3]. Compared to late‐stage patients, NSCLC patients diagnosed at an early stage have a better prognosis [4]. However, wide heterogeneity in overall survival has been observed even within the same stage of cancer, indicating the possible existence of prognosis‐influencing molecular mechanisms [5]. Epigenetic alterations such as DNA methylation are considered important representatives of these molecular mechanisms [6].

Tripartite motif containing 27 (*TRIM27*) is highly expressed in lung cancer and plays an important role in cancer prognosis [7, 8] by encoding a member of the tripartite motif (TRIM) family. *TRIM* family proteins play crucial roles in a wide range of processes, including cell growth, apoptosis and stem cell differentiation [9]. *TRIM27* is an oncogene of various tumour types, including colitis‐associated cancer, salivary gland intraductal carcinoma, colon cancer, uterus cancer and prostate cancer [7, 10, 11]. Further, originally identified to be involved in oncogenic rearrangements with the transfection proto‐oncogene (RET), *TRIM27* is also known as RFP (RET finger protein) [12]. RET rearrangements were implicated in NSCLC [13].

DNA methylation has been recognized as cancer biomarkers and therapeutic targets for NSCLC [14, 15, 16, 17, 18, 19, 20, 21], as well as other cancers [22, 23], since it is stable to measure but is modifiable with proper interventions [24, 25]. Also, aberrant DNA methylation of *TRIM27* was identified affecting lung function in monozygotic twins [26]. Nevertheless, the association between DNA methylation of *TRIM27* and early‐stage NSCLC survival still remains largely unclear.

Furthermore, DNA methylation changes have been linked to various environmental exposures (e.g., cigarette smoking) and may explain part of the association between smoking and cancer recurrence and mortality [15, 27, 28]. However, LUAD is more common in nonsmokers and long‐term former smokers, while most NSCLC patients among current smokers have LUSC [29, 30], indicating substantially different pathology and oncology. Anyway, few study focused on heterogeneous effect of DNA methylation between LUAD and LUSC.

Therefore, we utilized a two‐stage design to identify NSCLC prognosis associated epigenetic biomarkers in *TRIM27* and further explored the potential reason of heterogeneous effect of biomarkers across histology by performing epigenetic–smoking interaction analysis. Meanwhile, the robustly significant biomarkers were investigated for the associated alterations in gene expression which were also studied for effect on lung cancer survival.

## 2 Materials and methods

### 2.1 Study populations

We collected data from early‐stage (stage I and II) NSCLC patients from five international study centres. Cases from the Harvard, Spain, Norway and Sweden cohorts were assigned into the discovery phase [31, 32, 33, 34], while cases from The Cancer Genome Atlas (TCGA) were assigned into the validation phase. All patients provided written informed consent. The study methodologies conformed to the standards set by the Declaration of Helsinki and was approved by the local ethics committee.

#### 2.1.1 Harvard

The Harvard Lung Cancer Study cohort was described previously [31]. Patients were recruited at Massachusetts General Hospital (MGH) since 1992. All were newly diagnosed and histologically confirmed as primary NSCLC at the time of recruitment. Snap‐frozen tumour samples were taken from patients during complete resection. The 151 early‐stage patients selected in this study had complete survival information. Tumour DNA was extracted from 5‐μm‐thick histopathologic sections. Each specimen was evaluated by an MGH pathologist for amount (tumour cellularity > 70%) and quality of tumour cells. All specimens were histologically classified using World Health Organization criteria.

#### 2.1.2 Spain

The study population was described previously [32]. Tumours were collected by surgical resection from 226 patients. DNA extraction was performed on tumour specimens (10 μm thick, tumour cellularity > 50%). The study was approved by the Bellvitge Biomedical Research Institute Institutional Review Board.

#### 2.1.3 Norway

Participants were 133 LUAD patients with operable lung cancer tumours seen at Oslo University Hospital, Rikshospitalet, Norway, in 2006–2011 [33]. Tumour tissues were collected during surgery, snap‐frozen in liquid nitrogen, and stored at −80 °C until DNA isolation. All early‐stage patients did not receive chemotherapy or radiotherapy before surgery. The project was approved by the Oslo University Institutional Review Board and the Regional Ethics Committee (S‐05307).

#### 2.1.4 Sweden

Tumour tissue samples were collected from 103 patients with early‐stage NSCLC who underwent operation at Skane University Hospital, Lund, Sweden [34]. The study was approved by the Regional Ethical Review Board in Lund, Sweden (Registration no. 2004/762 and 2008/702).

#### 2.1.5 TCGA

TCGA dataset included 332 LUAD and 285 LUSC cases. Overall survival times and common covariates were included. Level‐1 HumanMethylation450 DNA methylation data (image data) of each patient were downloaded from https://portal.gdc.cancer.gov on 1 October 2015.

### 2.2 Quality control procedures for DNA methylation data

For each patient, DNA methylation was assessed using Infinium HumanMethylation450 BeadChips (Illumina Inc., San Diego, CA, USA). All centres followed the same quality control (QC) procedures before conducting the association study. GenomeStudio Methylation Module V1.8 (Illumina Inc.) was used to convert raw image data into beta values (continuous numbers ranging 0–1) for background subtraction and control normalization. Unqualified probes meeting any one of the following criteria were excluded: (a) failed detection (*P* > 0.05) in > 5% of samples; (b) coefficient of variance of < 5%; (c) all samples methylated or unmethylated; (d) common single nucleotide polymorphisms located in the probe sequence or 10‐bp flanking regions; (e) cross‐reactive probes or cross‐hybridizing probes [35]; or (f) did not pass QC in all centres. Samples with > 5% undetectable probes were excluded. Methylation signals were further processed for quantile normalization (betaqn function in r package minfi) as well as type I and II probe correction (BMIQ function in r package lumi). Data were adjusted for batch effects (ComBat function in r package sva) according to the best pipeline by a comparative study [36]. Details of QC processes are described in Fig. S1.

### 2.3 Gene expression data

In TCGA cohort, all of the 281 LUAD and 277 LUSC cases had complete mRNA sequencing data. Gene expression was measured by RNA sequencing. Data processing and QC were done by TCGA workgroup. Raw counts were normalized by RNA‐seq expectation maximization. Level‐3 gene quantification data were downloaded from TCGA and were further checked for quality. Expression of *TRIM27* was extracted and log_2_‐transformed before analysis.

### 2.4 Statistical analysis

**Fig. 1:**
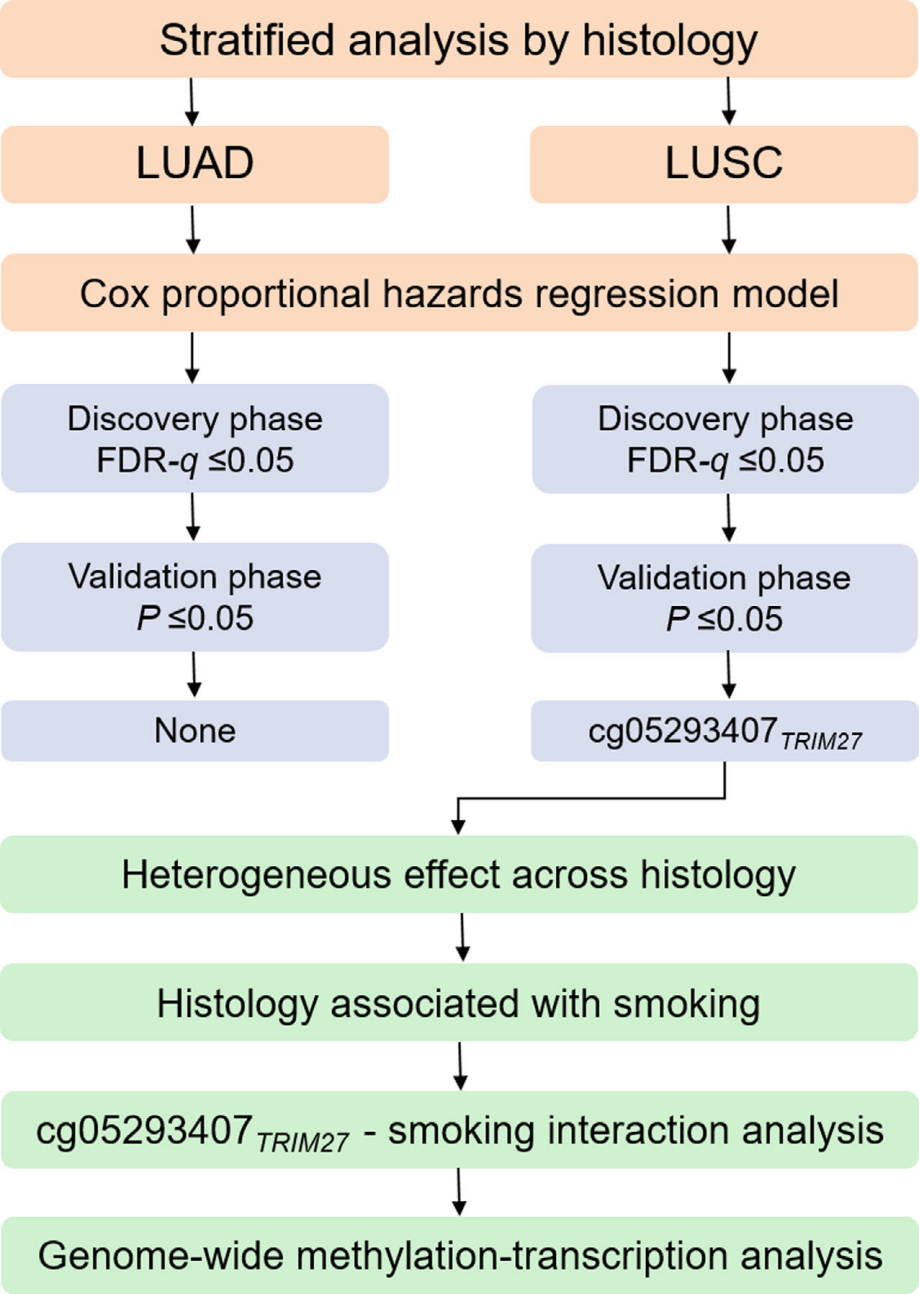
Flow chart of study design and statistical analyses.

The study design is shown in Fig. 1. To investigate the association between DNA methylation of *TRIM27* and overall survival, we applied a Cox proportional hazards model adjusted for age, sex, smoking status, clinical stage and study centre for LUAD and LUSC patients, respectively. Proportional hazards assumption for each CpG probe was also tested. Hazard ratio (HR) and 95% confidence interval (CI) were with respect to per 1% level of methylation increment. Multiple comparisons were adjusted by using false discovery rate method (FDR; measured by FDR*‐q* value) [37] to control the overall false‐positive rate at 5% level. CpG probes with FDR*‐q* ≤ 0.05 in the discovery phase were further replicated in the validation phase. Robustly significant CpG probes were finally retained if they met the following criteria: (a) *P* ≤ 0.05 in validation phase and (b) consistent effect direction across two phases. For robustly significant CpG probes, Kaplan–Meier curves were used to compare survival difference between patients with different methylation levels.

### 2.5 Methylation–smoking interaction analysis

We observed a significant heterogeneous effect of cg05293407*_TRIM27_* across histology, but the distributions of cg05293407*_TRIM27_* methylation in LUAD and LUSC patients were comparable. Meanwhile, combined prior literature information with our results, all evidence indicated that heavy smoking was relevant to LUSC. Therefore, we hypothesized that this heterogeneity might be explained by methylation–smoking interaction, which was further tested as a product term (methylation and pack‐year of smoking) in a Cox proportional hazards model adjusted for same covariates as aforementioned.

### 2.6 Genome‐wide methylation‐transcription analysis

Based on hypothesis of omnigenetic model [38], for these identified prognostic CpG probes, we used a linear regression model adjusted for the aforementioned covariates to test the association between DNA methylation and gene expression using transcriptomic data from TCGA. Significant genes were identified as FDR‐*q* ≤ 0.05 and presented in Circos plot. Then, the association between gene expression and overall survival was further evaluated using Cox models adjusted for the same covariates. Genes significantly associated with both methylation and NSCLC survival were screened out.

Continuous variables were expressed as mean ± standard deviation (SD), and categorical variables were expressed in frequency (*n*) and proportion (%). Statistical analysis was performed using r version 3.5.2 (The R Foundation of Statistical Computing, Tsinghua University, Beijing, China).

## 3 Results

Demographic and clinical information for patients with DNA methylation and gene expression data were detailed in Table 1 and Table S1. There were 96 CpG probes located in *TRIM27* (Table S2). In LUAD patients, none of the CpG probes in the discovery phase were identified by the criterion of FDR‐*q* ≤ 0.05 (Table S3). One probe, cg05293407*_TRIM27_*, was significantly associated with LUSC survival in both discovery (HR = 2.10, 95% CI: 1.41–3.12, *P* = 2.70 × 10^−4^, FDR‐*q* = 0.026) and validation phases (HR = 1.49, 95% CI: 1.07–2.07, *P* = 0.018) and showed robust association in combined data (HR = 1.65, 95% CI: 1.30–2.09, *P* = 4.52 × 10^−5^) (Tables S4 and S5).

**Table 1:** Demographic and clinical descriptions for early‐stage NSCLC patients in five international study centres.

Variable	Discovery					Validation	Combined
	USA (*N* = 151)	Spain^a^ (*N* = 226)	Norway (*N* = 133)	Sweden (*N* = 103)	All (*N* = 613)	TCGA (*N* = 617)	Overall (*N* = 1230)
Age (years)	67.67 ± 9.92	65.67 ± 10.58	65.52 ± 9.34	67.54 ± 9.99	66.44 ± 10.08	66.51 ± 9.47	66.48 ± 9.78
Sex							
Female	67 (44.37%)	105 (46.46%)	71 (53.38%)	54 (52.43%)	297 (48.45%)	255 (41.33%)	552 (44.88%)
Male	84 (55.63%)	121 (53.54%)	62 (46.62%)	49 (47.57%)	316 (51.55%)	362 (58.67%)	678 (55.12%)
Smoking status							
Never	18 (11.92%)	30 (13.57%)	17 (12.78%)	18 (17.48%)	83 (13.65%)	55 (9.18%)	138 (11.43%)
Former	81 (53.64%)	120 (54.30%)	74 (55.64%)	54 (52.43%)	329 (54.11%)	376 (62.77%)	705 (58.41%)
Current	52 (34.44%)	71 (32.13%)	42 (31.58%)	31 (30.10%)	196 (32.24%)	168 (28.05%)	364 (30.16%)
Unknown	0	5	0	0	5	18	23
Clinical stage							
I	104 (68.87%)	183 (80.97%)	93 (69.92%)	95 (92.23%)	475 (77.49%)	393 (63.70%)	868 (70.57%)
II	47 (31.13%)	43 (19.03%)	40 (30.08%)	8 (7.77%)	138 (22.51%)	224 (36.30%)	362 (29.43%)
Histology							
LUAD	96 (63.58%)	183 (80.97%)	133 (100.00%)	80 (77.67%)	492 (80.26%)	332 (53.81%)	824 (66.99%)
LUSC	55 (36.42%)	43 (19.03%)	0 (0.00%)	23 (22.33%)	121 (19.74%)	285 (46.19%)	406 (33.01%)
Chemotherapy							
No	142 (94.04%)	177 (90.77%)	102 (76.69%)	67 (90.54%)	488 (88.25%)	194 (76.98%)	682 (84.72%)
Yes	9 (5.96%)	18 (9.23%)	31 (23.31%)	7 (9.46%)	64 (11.75%)	58 (23.02%)	123 (15.28%)
Unknown	0	31	0	29	60	365	425
Radiotherapy							
No	132 (87.42%)	184 (94.36%)	132 (99.25%)	74 (100.00%)	522 (94.39%)	239 (94.84%)	761 (94.53%)
Yes	19 (12.58%)	11 (5.64%)	1 (0.75%)	0 (0.00%)	31 (5.61%)	13 (5.16%)	44 (5.47%)
Unknown	0	31	0	29	60	365	425
Adjuvant therapy^b^							
No	127 (84.11%)	168 (86.15%)	101 (75.94%)	67 (90.54%)	463 (83.73%)	187 (74.21%)	650 (80.75%)
Yes	24 (15.89%)	27 (13.85%)	32 (24.06%)	7 (9.46%)	90 (16.27%)	65 (25.79%)	155 (19.25%)
Unknown	0	31	0	29	60	365	425
Survival year^c^							
Median (95% CI)	6.66 (5.41–7.87)	7.12 (5.06–9.63)	7.36 (6.77–7.95)^*^	7.39 (4.98–9.12)	7.39 (6.50–8.23)	4.54 (3.68–5.41)	6.60 (5.84–7.35)
Censoring rate	19.21%	55.31%	68.42%	43.69%	43.31%	76.99%	62.20%

^a^ Spain centre is a collaborative study centre, containing samples from Spain, Italy, UK, France and United States.

^b^ Adjuvant therapy included chemotherapy and/or radiotherapy.

^c^ Restricted mean survival time was given because median was not available; proportion of samples lost to follow‐up or alive at end of study.

**Fig. 2:**
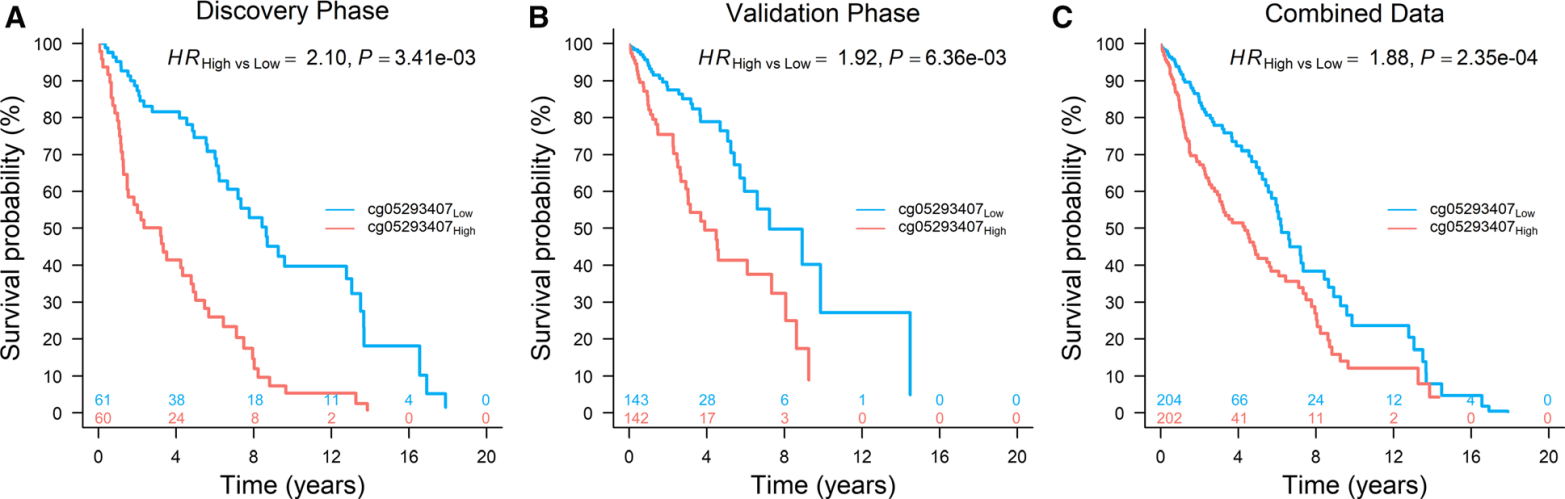
Kaplan–Meier survival curves of LUSC patients. High and low methylation groups were defined according to median value in (A) discovery phase (*N* = 121, median value: 1.72%), (B) validation phase (*N* = 285, median value: 1.48%) and (C) combined data (*N* = 406, median value: 1.55%). HR, 95% CI, and *P* value were derived from a Cox proportional hazards regression model adjusted for age, sex, smoking status, clinical stage and study centre.

**Fig. 3:**
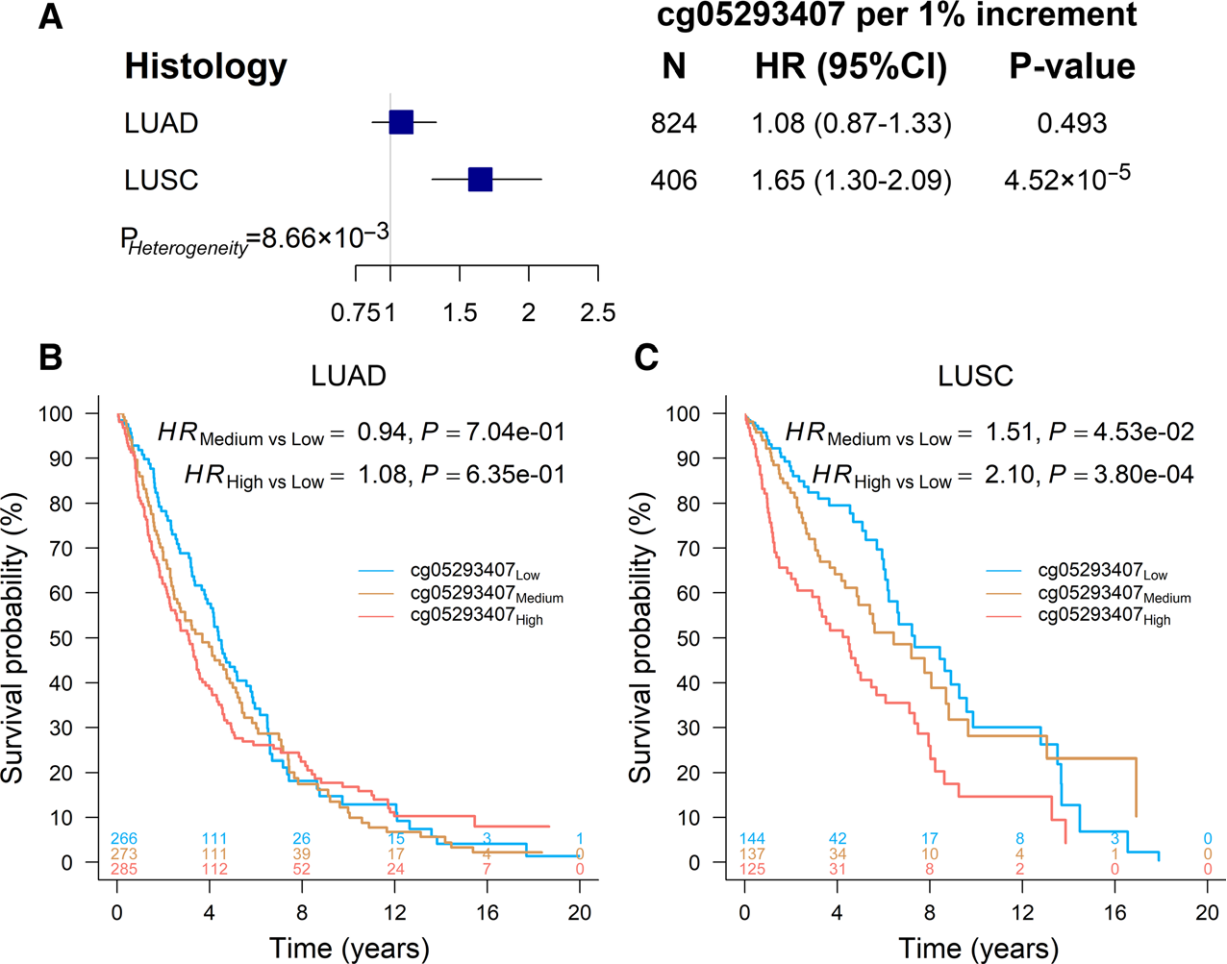
Methylation and histology interaction on survival of NSCLC patients. (A) Forest plot of the effects of cg05293407*_TRIM27_* in LUAD and LUSC populations. HR, 95% CI, and *P* value were derived from a Cox proportional hazards regression model adjusted for age, sex, smoking status, clinical stage and study centre. *P*_Heterogeneity_ was used to evaluate heterogeneity across both groups. (B) Kaplan–Meier survival curves of LUAD patients (*N* = 824) with high, medium and low methylation categorized by tertiles (1.38% and 1.84%) of cg05293407*_TRIM27_*. (C) Kaplan–Meier survival curves of LUSC patients (*N* = 406) with high, medium and low methylation categorized by tertiles (1.32% and 1.79%) of cg05293407*_TRIM27_*.

To further exemplify the effect of cg05293407*_TRIM27_* on overall survival, patients were categorized into two groups (high vs low) based on median value of methylation level. Kaplan–Meier survival curves showed significant differences between two groups in the discovery phase (HR_High vs Low_ = 2.10, 95% CI: 1.28–3.45, *P* = 3.41 × 10^−3^), the validation phase (HR_High vs Low_ = 1.92, 95% CI: 1.20–3.07, *P* = 6.36 × 10^−3^) and combined data (HR_High vs Low_ = 1.88, 95% CI: 1.34–2.63, *P* = 2.35 × 10^−4^) (Fig. 2). However, the effect of cg05293407*_TRIM27_* on LUAD survival was not significant (HR = 1.08, 95% CI: 0.87–1.33, *P* = 0.493) (Fig. 3A), indicating a significant heterogeneous effect of cg05293407*_TRIM27_* across NSCLC histology (*P* = 8.66 × 10^−3^). Also, the pattern of survival curves for patients with high, medium and low methylation levels based on tertiles of methylation level differed between LUAD and LUSC patients. Survival curves were significantly separated only in LUSC patients (Fig. 3B,C).

**Fig. 4:**
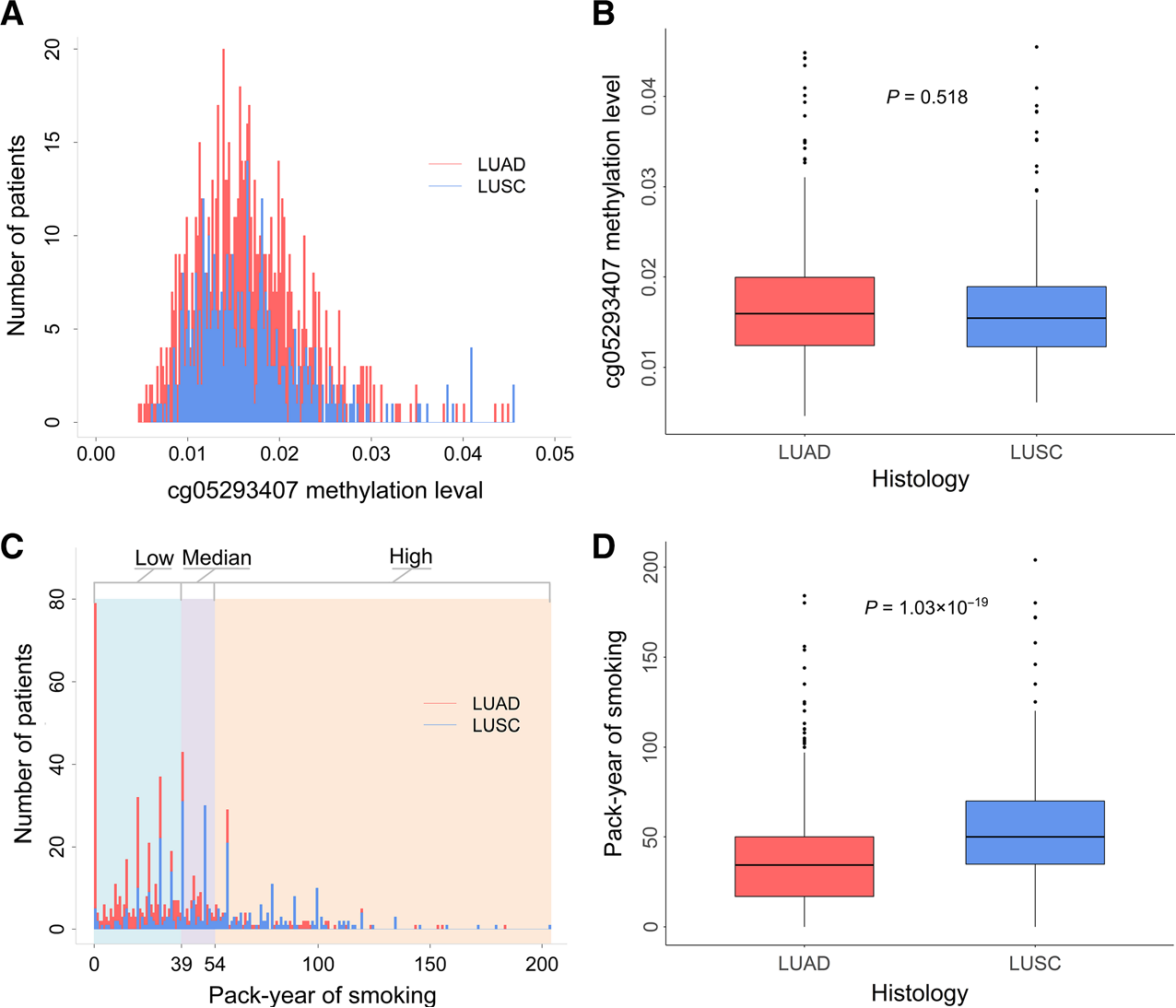
Distribution of cg05293407*_TRIM27_* and pack‐year of smoking in LUAD (*N* = 824) and LUSC patients (*N* = 406). (A) Distribution of cg05293407*_TRIM27_*. (B) Box plot of cg05293407*_TRIM27_*. (C) Distribution of pack‐year of smoking. And, 39 and 54 are the two cut‐off values of the three groups of pack‐year of smoking (low, medium and high). (D) Box plot of pack‐year of smoking in LUAD patients (*N* = 652) and LUSC patients (*N* = 342). Wilcoxon rank‐sum test was used to estimate the *P* value.

However, distribution of cg05293407*_TRIM27_* methylation in LUAD and LUSC patients was similar (Fig. 4A) and comparable (*P* = 0.518) by Wilcoxon rank‐sum test (Fig. 4B). Since nonsmokers and long‐term former smokers are more common in LUAD patients, while the majority of lung cancer patients who are current smokers have LUSC [29]. Therefore, we assumed that there might exist a methylation–smoking interaction accounting for the heterogeneous effect of cg05293407*_TRIM27_* on NSCLC survival across histology. The smoking‐related variables were compared between the LUAD and LUSC patients (Table S6). Compared with LUAD patients, LUSC patients had more pack‐year of smoking averagely (37.49_LUAD_ vs 54.79_LUSC_, *P* = 1.03 × 10^−19^) (Fig. 4C,D) and a higher proportion of current smokers (28.24%_LUAD_ vs 34.09%_LUSC_, *P* = 0.037) (Table S6).

**Fig. 5:**
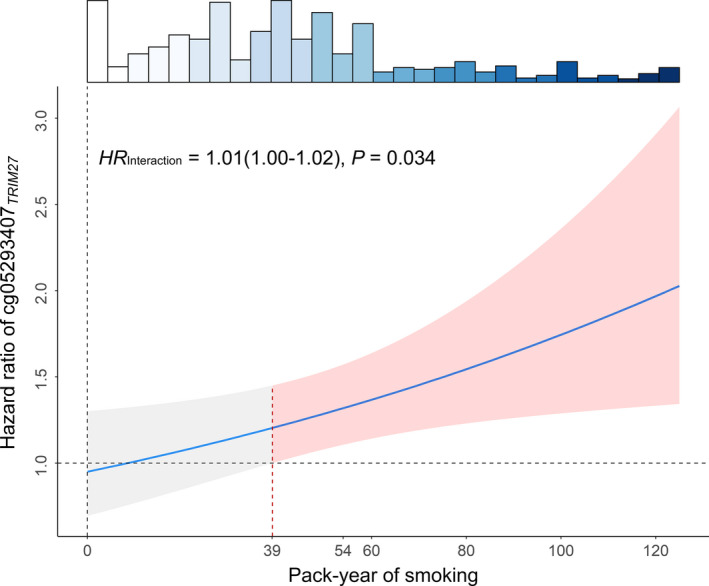
Methylation–smoking interaction on survival of NSCLC patients (*N* = 994). HR of interaction term, 95% CI, and *P* value were derived from a Cox proportional hazards regression model adjusted for age, sex, smoking status, clinical stage, cancer type and study centre. HR of methylation estimated based on level of pack‐year of smoking. Shallow area represented 95% CI. Histogram on the top shows the distribution of pack‐year of smoking.

We identified a significant interaction between cg05293407*_TRIM27_* and pack‐year of smoking in all NSCLC patients (HR_interaction_ = 1.01, 95% CI: 1.00–1.02, *P* = 0.034). With increased pack‐year of smoking, there was an elevated risk for high methylation of cg05293407*_TRIM27_* on NSCLC survival (Fig. 5). Therefore, pack‐year of smoking was a modifier of the association between cg05293407*_TRIM27_* and NSCLC survival.

We also evaluated joint effect of cg05293407*_TRIM27_* methylation level and pack‐year of smoking on NSCLC survival (Table 2). Patients were categorized into three groups (high, medium and low) by tertiles of cg05293407*_TRIM27_* methylation level (1.33% and 1.78%) and were also categorized into three groups (high, medium and low) by cut‐off values of pack‐year of smoking (39 and 54). Only for these patients having > 39 pack‐year of smoking, cg05293407*_TRIM27_* was a significant risk factor (Fig. 5). Therefore, 39 was defined as a cut‐off value of low and medium–high levels. Further, the median value (54) of pack‐year of smoking for LUAD patients having > 39 pack‐year of smoking was used to distinguish medium and high levels. We used the best prognosis group (low–medium methylation of cg05293407*_TRIM27_* and low–medium pack‐year of smoking) as the reference to evaluate effects of high methylation level, high pack‐year of smoking and their joint effect, as well as interaction. In the combined dataset, the main effect of high pack‐year of smoking was HR = 1.12 (95% CI: 0.84–1.49; *P* = 0.438), and the main effect of high methylation of cg05293407*_TRIM27_* was HR = 1.10 (95% CI: 0.83–1.45; *P* = 0.502). However, the joint effect was HR = 2.00 (95% CI: 1.43–2.79; *P* = 5.00 × 10^−5^), which was greater than the product of the two individual risk effects (1.2320 = 1.12 × 1.10), indicating a synergistic interaction between high methylation of cg05293407*_TRIM27_* and high pack‐year of smoking (HR_interaction_ = 1.62; 95% CI: 1.04–2.53; *P* = 3.21 × 10^−2^).

**Table 2:** Joint effect and interaction of elevated methylation and pack‐year of smoking on prognosis of early‐stage NSCLC.

Effect type ^a^	High methylation^b^	High pack‐year of smoking	Number	Death	Crude mortality	HR (95% CI) ^a^	*P* ^a^
	No	No	486	158	32.51%	Reference	
Main effect _1_	No	Yes	177	77	43.50%	1.12 (0.84, 1.49)	0.438
Main effect _2_	Yes	No	229	94	41.05%	1.10 (0.83, 1.45)	0.502
Joint effect	Yes	Yes	102	56	54.90%	2.00 (1.43, 2.79)	5.00 × 10^−5^
Interaction ^c^						1.62 (1.04, 2.53)	3.21 × 10^−2^

^a^ Patients were categorized into three groups (high vs medium–low) by tertiles of cg05293407*_TRIM27_* methylation level (1.33% and 1.78%) and were categorized into three groups (high vs medium–low) by cut‐off value of pack‐year of smoking (39 and 54). The risk effect of cg05293407*_TRIM27_* on survival was significant only for these patients having > 39 pack‐year of smoking. And, 54 was the median value of pack‐year of smoking for LUAD patients having > 39 pack‐year of smoking.

^b^ Main effects of elevated methylation and pack‐year of smoking as well as their joint effect and interaction were derived from Cox proportional hazards regression model adjusted for age, sex, clinical stage, histology, study centre, and stratified by cancer type.

^c^ Interaction = Joint effect ÷ (Main effect _1_ × Main effect _2_). 1.62 ≈ 2.00 ÷ (1.12 × 1.10).

**Fig. 6:**
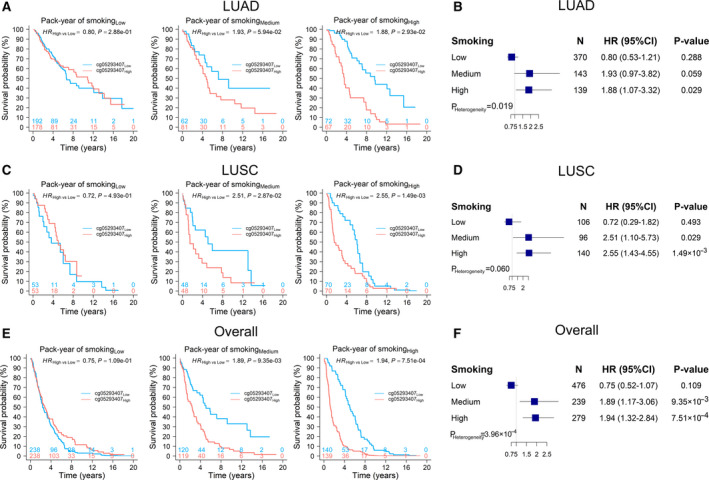
Kaplan–Meier survival curves of LUAD, LUSC and overall NSCLC patients. Kaplan–Meier curves of low and high methylation groups and forest plots of the effects of cg05293407*_TRIM27_* were stratified by populations having low, medium and high pack‐year of smoking for (A, B) LUAD patients (*N*_Low_ = 370, *N*_Medium_ = 143, *N*_High_ = 139), (C, D) LUSC patients (*N*_Low_ = 106, *N*_Medium_ = 96, *N*_High_ = 140) and (E, F) overall patients (*N*_Low_ = 476, *N*_Medium_ = 239, *N*_High_ = 279). HR, 95% CI, and *P* value were derived from a Cox proportional hazards regression model adjusted for age, sex, smoking status, clinical stage and study centre. *P*_Heterogeneity_ was used to evaluate heterogeneity of HRs across groups.

To illustrate the modification effect by pack‐year of smoking, effect of cg05293407*_TRIM27_* on NSCLC survival was evaluated in patients with low, medium and high levels of pack‐year of smoking. The effect of cg05293407*_TRIM27_* varied across patients with different pack‐year of smoking. For LUAD patients with a high level of pack‐year of smoking, high methylation of cg05293407*_TRIM27_* had significantly worse survival (HR_High vs Low_ = 1.88, 95% CI: 1.07–3.32, *P* = 0.029) (Fig. 6A,B). In LUSC and overall NSCLC patients, we observed similar significant results in patients with both medium (HR_High vs Low_ = 2.51, 95% CI: 1.10–5.73, *P* = 0.029 in LUSC patients; HR_High vs Low_ = 1.89, 95% CI: 1.17–3.06, *P* = 9.35 × 10^−3^ in overall patients) and high (HR_High vs Low_ = 2.55, 95% CI: 1.43−4.55, *P* = 1.49 × 10^−3^ in LUSC patients; HR_High vs Low_ = 1.94, 95% CI: 1.32–2.84, *P* = 7.51 × 10^−4^ in overall patients) levels of pack‐year of smoking (Fig. 6C–F). Our results indicated that cg05293407*_TRIM27_* influenced NSCLC survival actually regardless of histology, but only among these patients exposed to relatively heavy smoking. Since the pack‐year of smoking might be bimodal distributed due to plenty of zero values from never smokers, we also performed sensitivity analysis by testing the methylation–smoking interaction in NSCLC patients excluding never smokers and still observed the significant interaction (Fig. S2) and same pattern (Fig. S3). Another sensitivity analysis based on smoking status also indicated an upward trend (*P*_Trend_ = 0.022) in effect size of cg05293407*_TRIM27_* from never smokers (HR = 0.89), former smokers (HR = 1.23) to current smokers (HR = 1.88) in overall population, even not taking pack‐year of smoking into account (Fig. S4).

**Fig. 7:**
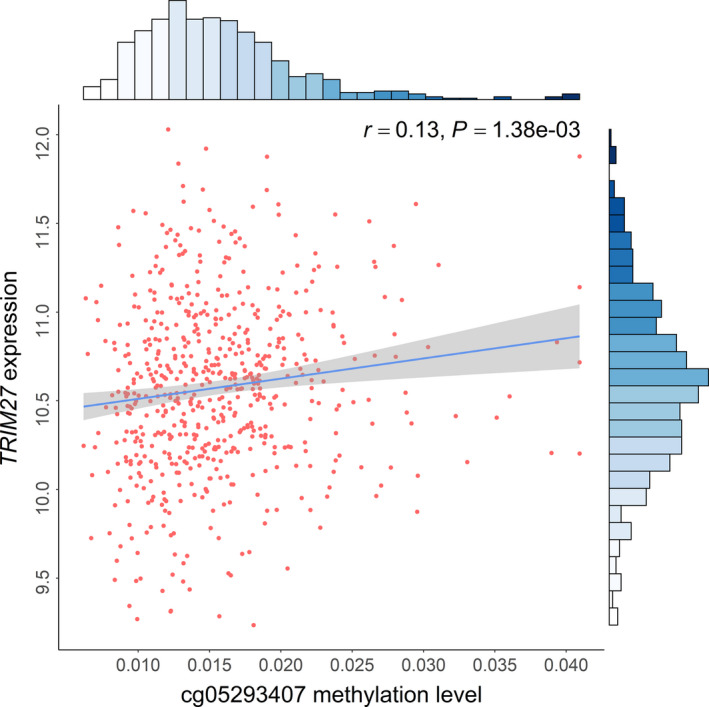
Association between DNA methylation of cg05293407 and expression of corresponding gene *TRIM27* in TCGA using 277 biologically independent samples. Correlation coefficients and hypothesis tests were based on Pearson correlation test. Gene expression was log2‐transformed before analysis. Histogram on top shows the distribution of cg05293407 methylation; histogram on side shows the distribution of *TRIM27* expression.

Further, because cg05293407*_TRIM27_* maps to *TRIM27*, the association between *TRIM27* expression and cg05293407*_TRIM27_* methylation was evaluated in the TCGA population. In NSCLC patients, there was a significant association (*r* = 0.13, *P* = 1.38 × 10^−3^) (Fig. 7), suggesting that methylation of cg05293407*_TRIM27_* upregulated *TRIM27* expression. However, *TRIM27* expression had no significant association with overall survival (HR = 0.84, 95% CI: 0.62–1.14, *P* = 0.270) (Fig. S5). However, genome‐wide methylation‐transcription analysis showed that expression of 29 genes was associated with cg05293407*_TRIM27_* (Table S7 and Fig. S6A). Among them, expression of three genes was associated with overall survival: ubiquitin‐specific protease 26 (*USP26*), gap junction protein gamma 3 (*GJC3*), and N‐acetylated alpha‐linked acidic dipeptidase 2 (*NAALAD2*) (Fig. S6B–D).

## 4 Discussion

We performed a two‐stage study and integrative analysis of DNA methylation of *TRIM27* and gene expression in early‐stage NSCLC patients. The CpG probe, cg05293407*_TRIM27_*, located at the 200 kb transcription start site (TSS) region of *TRIM27*, was identified as an exclusive biomarker of early‐stage LUSC prognosis. Further, the heterogeneous effect of cg05293407*_TRIM27_* across histology may be explained by a methylation–smoking interaction.

As LUAD and LUSC differ in the origin and histology, the mechanism of occurrence and progression may be different at a molecular level [39, 40]. For example, both mutated genes and recurrent somatic copy number alterations are largely distinct between the two NSCLC types [41]. We only observed one probe, cg05293407*_TRIM27_*, exclusively associated with early‐stage LUSC prognosis in stratified analysis by histology, whereas no promising CpG probes were observed for LUAD, possibly due to underlying epigenetic heterogeneity between LUAD and LUSC. Further, LUSC is more strongly associated with smoking than LUAD, suggesting different causes for their induction as well [30]. In addition, a methylation–smoking interaction may potentially provide interpretation of the heterogeneous effect of cg05293407*_TRIM27_*.

The tumour‐specific shift to transcriptional repression is associated with DNA methylation at TSSs in multiple tumour types [42]. Generally, hyper‐methylation blocks transcription initiation and reduces gene expression [43]. However, a small proportion of methylation surrounding the TSS region upregulates gene expression, indicating that DNA methylation regulation may be more complex [44]. In our study, DNA methylation at cg05293407 in the 200 kb TSS region of *TRIM27* upregulated gene expression in tumour tissues, which was consistent with previous reports [45, 46]. This phenomenon may be mediated by affecting the binding activity of upstream transcription factors [47]. However, further functional studies are warranted to elaborate the possible mechanism.

In LUSC patients, the methylation level of cg05293407*_TRIM27_* ranged from 0.62% to 4.09% and its median value was 1.48%, indicating a narrow range and low average level. As shown in Fig. S7, these maximum values of all 311 891 CpG probes followed a bimodal distribution with the first peak around 5%. Furthermore, there were 9341 (2.99%) CpG probes with even lower maximum value than that of cg05293407*_TRIM27_* indicating its narrow range was reasonable. Meanwhile, plenty of studies have revealed that aberrant DNA methylations of these hypo‐methylated CpG probes were also involved in diseases (e.g., female panic disorder risk associated cg07308824*_HECA_* and paediatric medulloblastoma prognosis associated cg02257300*_ERCC2_*) [48, 49].

**Fig. 8:**
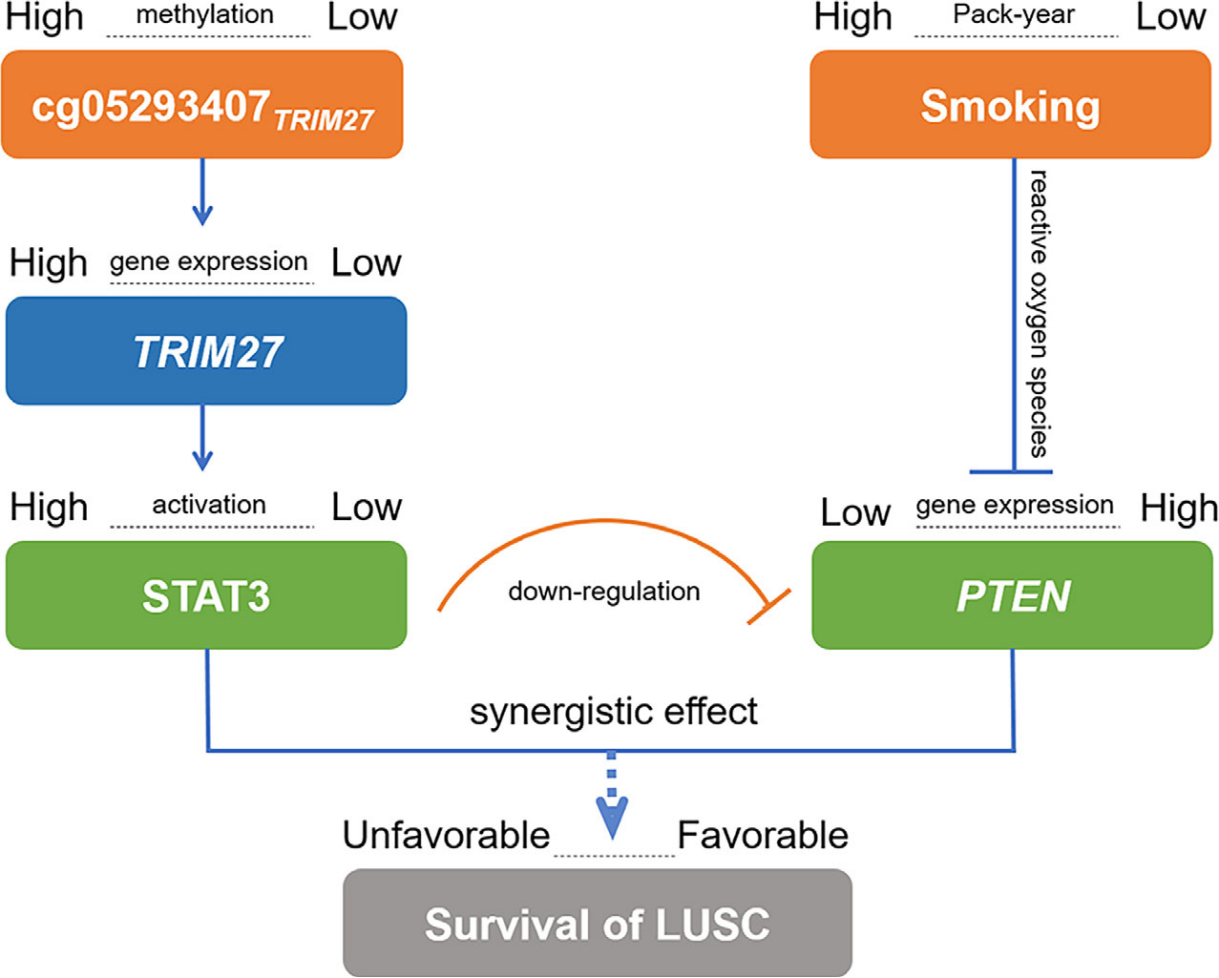
Diagram for pathway of DNA methylation–smoking interaction effect on survival for LUSC patients.

*TRIM27* belongs to the *TRIM* family, an extended family of proteins with a common denominator of a tripartite combinatorial motif encompassing RING finger, B‐box, and coiled‐coil domain homologies [50]. *TRIM27* is an important positive regulator of signal transducer and activator of transcription 3 (STAT3) activation. TRIM27, located at retromer‐positive structures, can recruit STAT3 after IL‐6 stimulation and lead to improved STAT3 activation [11]. STAT3 activity plays important roles in pathogenesis of many cancers, including breast, head and neck, prostate and brain cancers [51]. Further, STAT3 is overexpressed in NSCLC tumour samples, and sorafenib can inhibit STAT3 activation to produce anticancer effects in NSCLC [52]. Combined with our results, these data suggested that high methylation of cg05293407*_TRIM27_* might promote *TRIM27* expression, further leading to STAT3 activation and poor prognosis (Fig. 8).

Smoking is associated with several genetic alterations in NSCLC [53] and has been well‐established as a relevant factor of lung cancer risk as well as prognosis [15]. Cigarette smoke contains reactive oxygen species (ROS), which inhibit phosphatase and tensin homolog (*PTEN*) expression by phosphorylating the ROS‐dependent Src/EGFR‐p38MAPK pathway [54]. *PTEN* inhibits glycolysis in brain tumour cells by directly interacting with phosphoglycerate kinase 1 (PGK1) [55]. Further, *PTEN* inhibits cancer cells by moderating signalling through the PI3K pathway. *PTEN* is lowly expressed in NSCLC tumour samples and is more prevalent in LUSC [56]. Therefore, for patients with high pack‐year of smoking, heavy exposure to cigarette smoking may strongly inhibit *PTEN* expression through ROS and relate to poor NSCLC prognosis (Fig. 8).

Moreover, *PTEN* is an essential modulator of STAT3‐mediated pathways. Although STAT3 is a downstream target of *PTEN*, STAT3 also reversely inhibits *PTEN* expression by directly activating miR‐21, which is part of the epigenetic switch linking inflammation to cancer [57]. Therefore, STAT3 activation can downregulate *PTEN* expression (Fig. 8). In terms of the cg05293407*_TRIM27_* and smoking interaction, high methylation was associated with poor prognosis in NSCLC patients with medium–high pack‐year of smoking rather than low pack‐year of smoking, possibly because high activation of STAT3 and low expression of *PTEN* may only occur in patients with medium–high methylation of cg05293407*_TRIM27_*.

We observed three genes associated with cg05293407*_TRIM27_*: *GJC3*, *NAALAD2* and *USP26*. *GJC3* is one of the genes coding for connexin (CX) proteins and is reported to be associated with nonsyndromic hearing loss [58]. Further, patients with low *GJC3* expression had a better prognosis in our study. *NAALAD2* encodes human prostate‐specific membrane antigen (PSM), which is a marker of prostatic carcinomas and is the first shown to possess NAALADase activity [59]. Similarly, LUSC patients with lower *NAALAD2* expression had higher survival in our study. *USP26* is associated with Sertoli cell‐only syndrome and male infertility in both European and Chinese men [60, 61]. Further, our study showed consistent results in LUSC patients. Although these three genes lack explicit evidence of association with LUSC, their relationship to cg05293407*_TRIM27_* and LUSC survival may inspire functional studies of these potential genes and further help elucidate the mechanistic pathway of cg05293407*_TRIM27_* on LUSC survival.

Our study has several strengths. First, to our knowledge, this is the first multicentre study of interaction between DNA methylation of *TRIM27* and smoking, which attempted to interpret the effect of DNA methylation that varied by NSCLC histology. Second, besides the significant statistical interaction observed on a population level, we experimentally elaborated on a plausible functional interaction between two pathways based on literature evidence. Third, by controlling false positives, our two‐stage study and the sensitivity analysis provided robustness to our results. Fourth, we performed integrative analysis of DNA methylation and gene expression and systematically evaluated associated genes of cg05293407*_TRIM27_* on genome‐wide scale.

We also acknowledge some limitations. First, though three genes associated with cg05293407*_TRIM27_* further affected lung cancer prognosis in our study, there was no explicit evidence of their mechanisms. Therefore, these associations should be interpreted with caution. Second, with a high censoring rate in the TCGA cohort, the statistical power might be limited. Anyway, the association between cg05293407*_TRIM27_* and prognosis remained significant in TCGA, indicating our results were conservative and roust. Third, the positive association between cg05293407*_TRIM27_* and *TRIM27* expression was not reported by the other literatures yet. Further functional experiments are warranted to confirm our results. Finally, as the majority of our population was Caucasian (89.19%), generalization of the results to the other ethnicity groups should be cautioned.

## 5 Conclusion

In summary, our study identified cg05293407*_TRIM27_* as a potential biomarker for LUSC prognosis and laid out a case that the methylation–smoking interaction may account for heterogeneous effects of cg05293407*_TRIM27_* across histology. Our findings provide a potential dynamic and reversible therapeutic target for NSCLC patients.

## Conflict of interest

The authors declare no conflict of interest.

## Author contributions

XJ, LL, RZ, YW, FC and DCC contributed to the study design. RZ, LS, AS, MMB, AK, MP, JS, ÅH, ME and DCC contributed to data collection. XJ, LL, SS, XD, CC, YL, RZ, YW, FC and DCC performed statistical analysis and interpretation and drafted the manuscript. YZ, HH, XC, JC, LW, JH, WD, LS, YJ, JF, JG and DY revised the manuscript. All authors contributed to critical revision of the manuscript and approved its final version. Financial support and study supervision were provided by RZ, YW, FC and DCC.

## Supporting information


**Fig. S1.** Quality control processes for DNA methylation chip data.

**Fig. S2.** Methylation–smoking interaction on survival of LUSC patients excluding never smokers.

**Fig. S3.** Kaplan–Meier overall survival (OS) curves of LUAD, LUSC and overall NSCLC patients excluding never smokers. (A,B) LUAD patients, (C,D) LUSC patients and (E,F) overall patients. Hazard ratio (HR) and *P* value were derived from a Cox proportional hazards regression model adjusted for age, sex, smoking status, clinical stage, and study centre. *P*_Heterogeneity_ was used to evaluate heterogeneity of HRs across groups.

**Fig. S4.** Kaplan–Meier overall survival (OS) curves of LUAD, LUSC and overall NSCLC patients. (A,B) LUAD patients, (C,D) LUSC patients and (E,F) overall patients. Hazard ratio (HR) and *P* value were derived from a Cox proportional hazards regression model adjusted for age, sex, clinical stage, pack‐year of smoking and study centre. *P*_Trend_ was used to evaluate trend of HRs across groups.

**Fig. S5.** Kaplan–Meier overall survival (OS) curves of TCGA cases by low or high *TRIM27* expression. The gene expression divided into low and high groups by median value (10.26). Hazard ratio (HR) and *P* value were derived from a Cox proportional hazards regression model adjusted for age, sex, smoking status, clinical stage, and study centre.

**Fig. S6.** Genome‐wide methylation transcription analysis of LUSC patients from the TCGA cohort. (A) Circos plot of genome‐wide gene expression. For plots in B–D, left panels show correlation of (B) *USP26*, (C) *GJC3* or (D) *NAALAD2* expression (X‐axis) with methylation level at cg05293407*_TRIM27_* (Y‐axis). Right panels show Kaplan–Meier survival plots of gene expression divided into low and high groups by median value.

**Fig. S7.** Distribution of maximum value of all 311891 CpG probes in non‐small‐cell lung cancer (NSCLC) patients.

**Table S1.** Demographic and clinical characteristics of early‐stage NSCLC patients with gene expression data derived from TCGA.
**Table S2.** Annotation information for 96 CpG probes located in *TRIM27*.
**Table S3.** Results of association analysis of 96 DNA methylation probes of *TRIM27* in LUAD samples.
**Table S4.** Results of association analysis of 96 CpG probes of *TRIM27* in LUSC samples.
**Table S5.** Results of proportional hazards test for 96 CpG probes of *TRIM27* in LUSC samples.
**Table S6.** Comparison of smoking‐related characteristics of former and current smokers between early‐stage LUAD and LUSC.
**Table S7.** Results of genome‐wide methylation transcription analysis of 29 genes significantly associated with cg05293407 in TCGA LUSC samples.

## Data Availability

The DNA methylation image data of Harvard, Spain, Norway and Sweden study cohort can be requested from DCC, ME, ÅH and JS, respectively. Alternatively, it can be retrieved from gene expression omnibus database (GSE39279, GSE66836 and GSE56044). TCGA: https://tcga‐data.nci.nih.gov; now hosted at GDC: https://portal.gdc.cancer.gov.
