# Supplementary figures and images for: Epigenetic–smoking interaction reveals histologically heterogeneous effects of TRIM27 DNA methylation on overall survival among early‐stage NSCLC patients
Source: Mol Oncol. 2020 Sep 3;14(11):2759–74. doi: 10.1002/1878-0261.12785 (PMC7607178; doi:10.1002/1878-0261.12785)

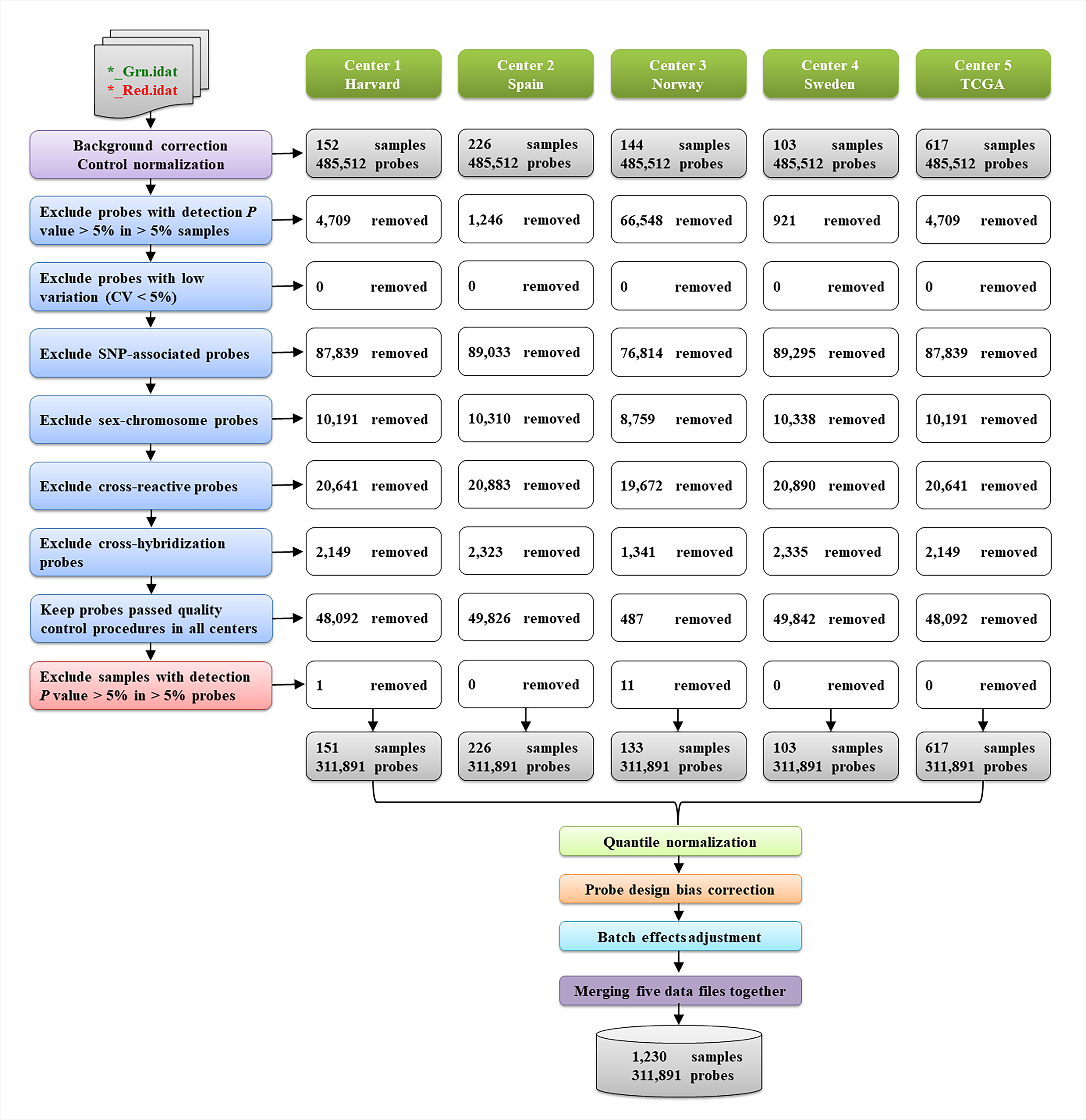

Supplement: Supplementary file 1 — Fig. S1. Quality control processes for DNA methylation chip data. [file MOL2-14-2759-s001.tif]

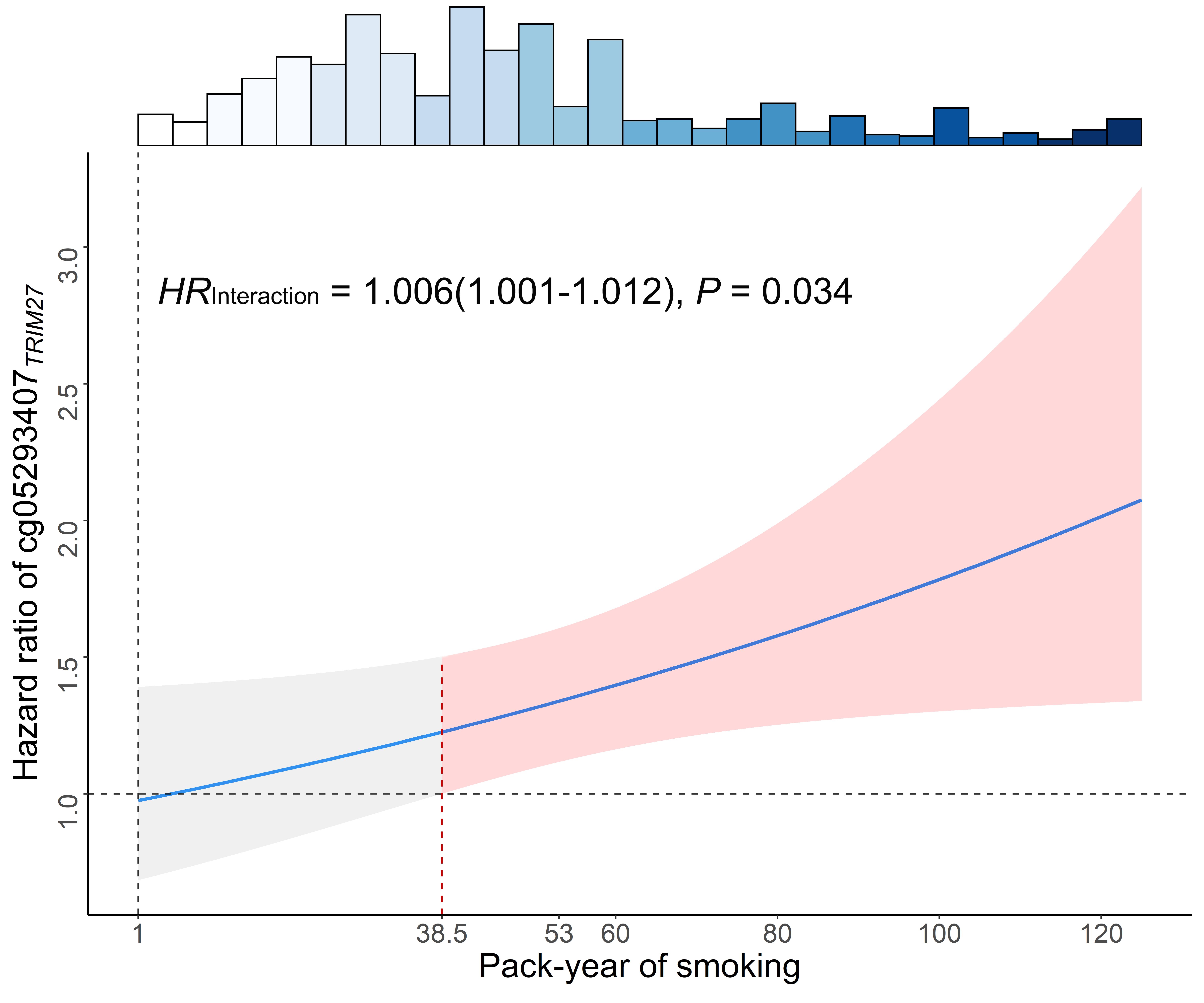

Supplement: Supplementary file 2 — Fig. S2. Methylation–smoking interaction on survival of LUSC patients excluding never smokers. [file MOL2-14-2759-s002.tif]

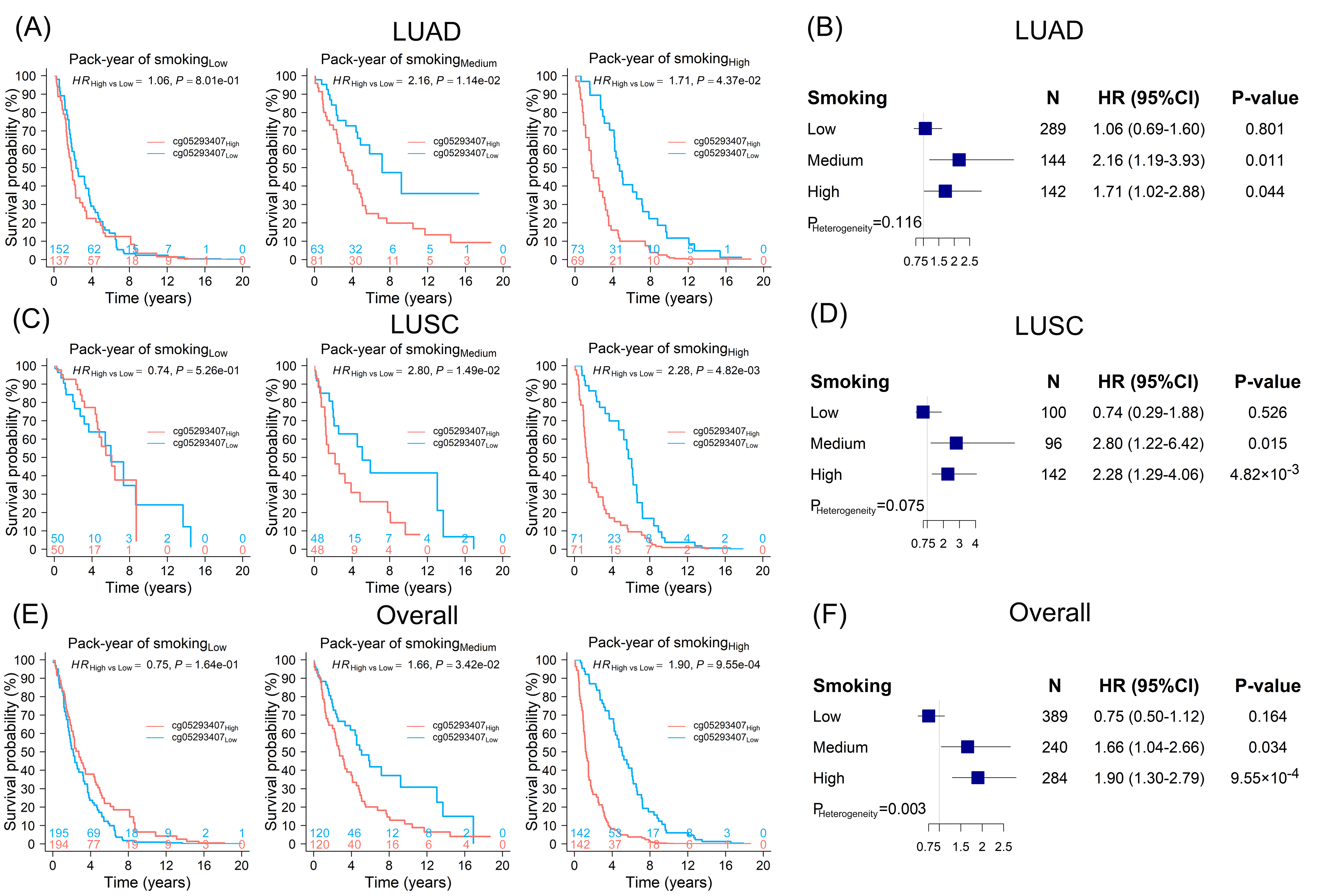

Supplement: Supplementary file 3 — Fig. S3. Kaplan–Meier overall survival (OS) curves of LUAD, LUSC and overall NSCLC patients excluding never smokers. (A,B) LUAD patients, (C,D) LUSC patients and (E,F) overall patients. Hazard ratio (HR) and P value were derived from a Cox proportional hazards regression model adjusted for age, sex, smoking status, clinical stage, and study centre. P Heterogeneity was used to evaluate heterogeneity of HRs across groups. [file MOL2-14-2759-s003.tif]

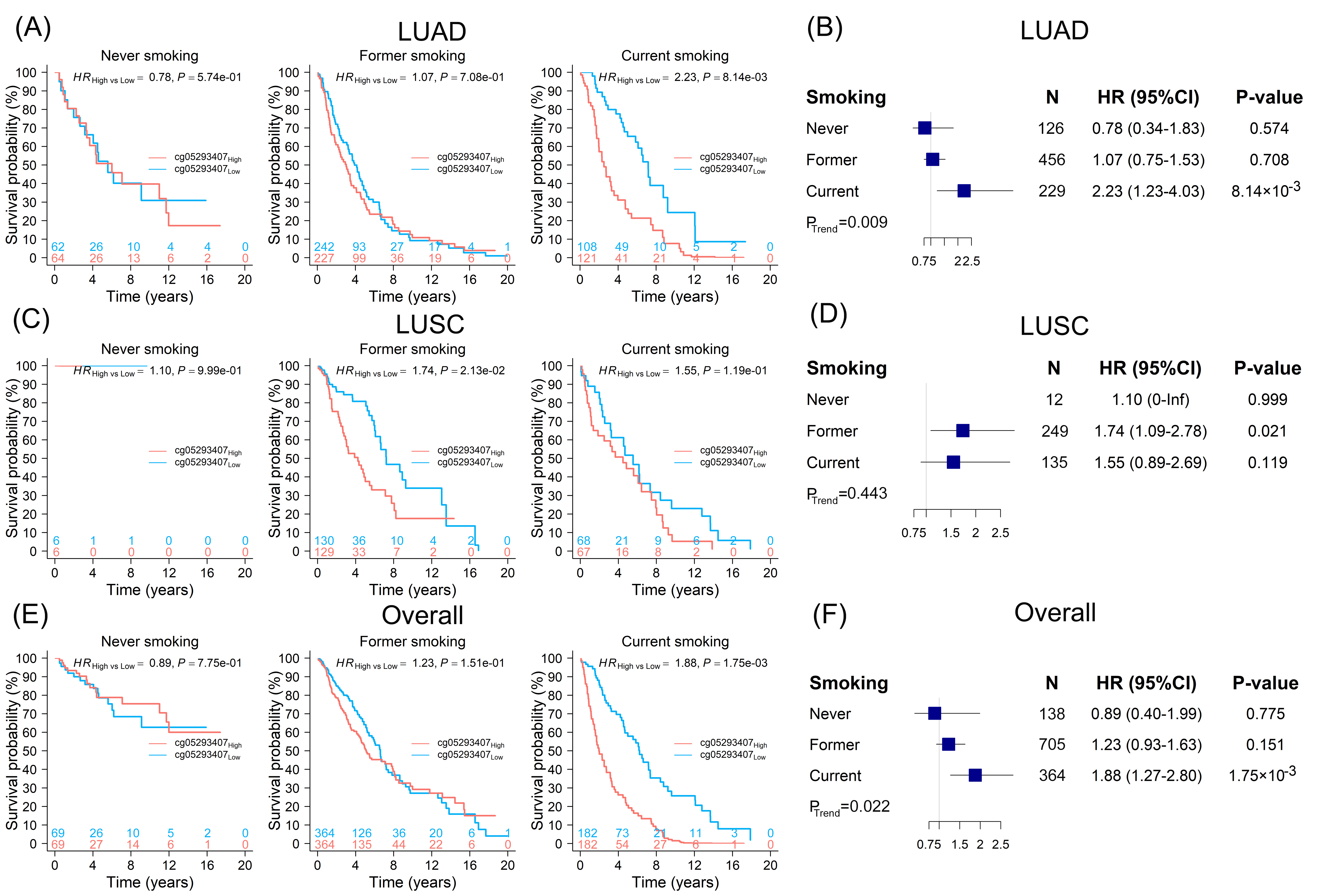

Supplement: Supplementary file 4 — Fig. S4. Kaplan–Meier overall survival (OS) curves of LUAD, LUSC and overall NSCLC patients. (A,B) LUAD patients, (C,D) LUSC patients and (E,F) overall patients. Hazard ratio (HR) and P value were derived from a Cox proportional hazards regression model adjusted for age, sex, clinical stage, pack‐year of smoking and study centre. P Trend was used to evaluate trend of HRs across groups. [file MOL2-14-2759-s004.tif]

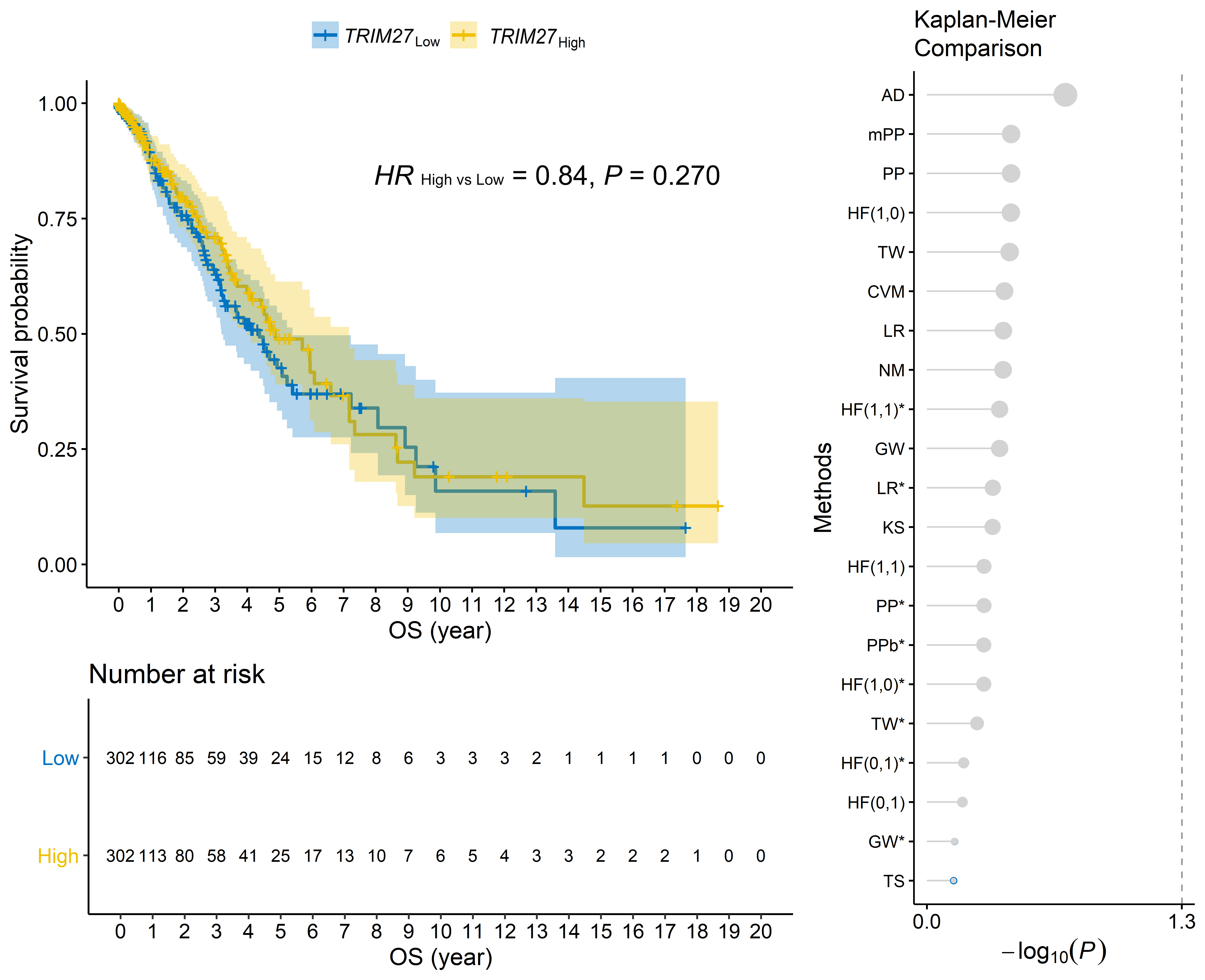

Supplement: Supplementary file 5 — Fig. S5. Kaplan–Meier overall survival (OS) curves of TCGA cases by low or high TRIM27 expression. The gene expression divided into low and high groups by median value (10.26). Hazard ratio (HR) and P value were derived from a Cox proportional hazards regression model adjusted for age, sex, smoking status, clinical stage, and study centre. [file MOL2-14-2759-s005.tif]

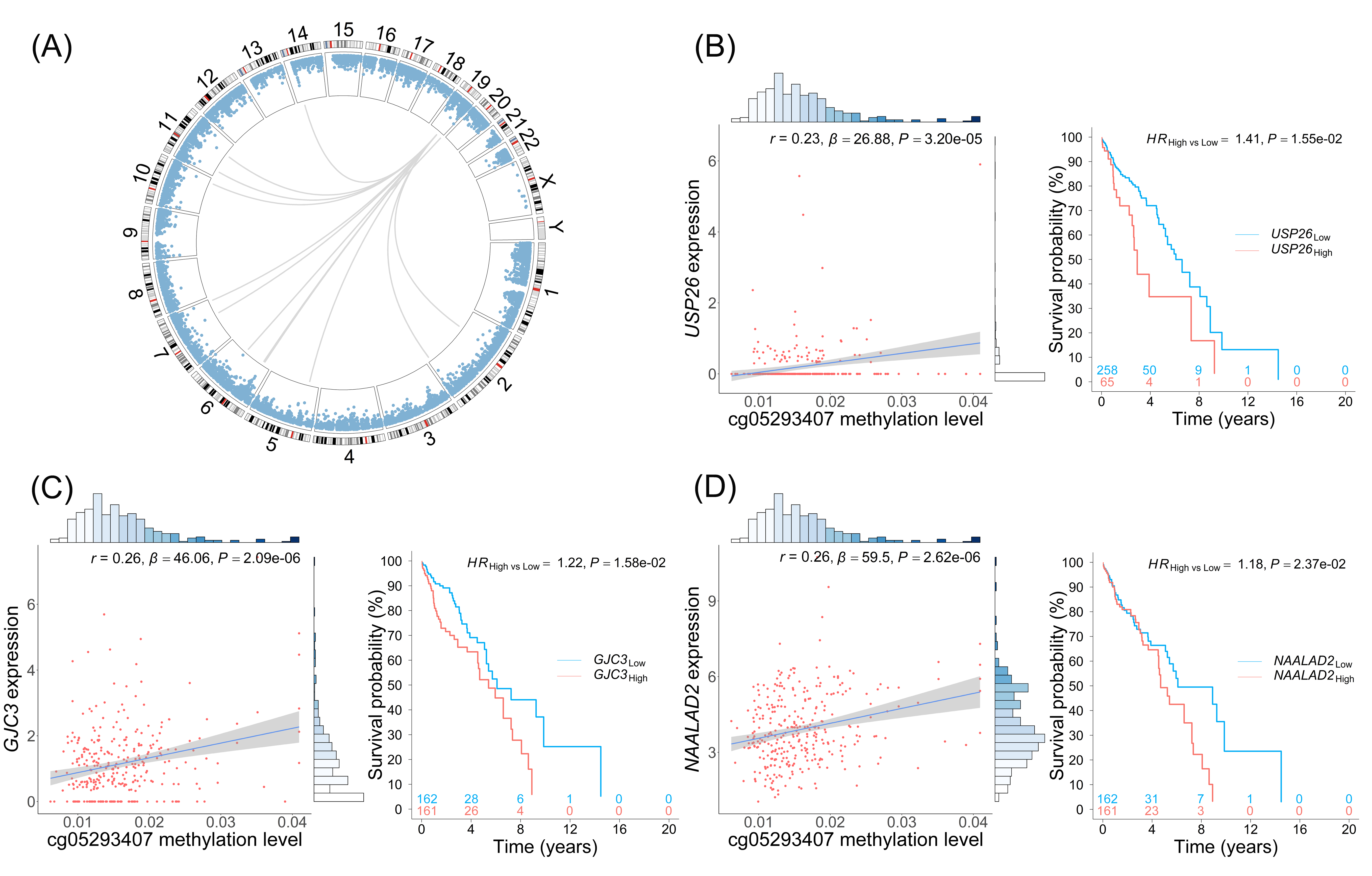

Supplement: Supplementary file 6 — Fig. S6. Genome‐wide methylation transcription analysis of LUSC patients from the TCGA cohort. (A) Circos plot of genome‐wide gene expression. For plots in B–D, left panels show correlation of (B) USP26, (C) GJC3 or (D) NAALAD2 expression (X‐axis) with methylation level at cg05293407TRIM27 (Y‐axis). Right panels show Kaplan–Meier survival plots of gene expression divided into low and high groups by median value. [file MOL2-14-2759-s006.tif]

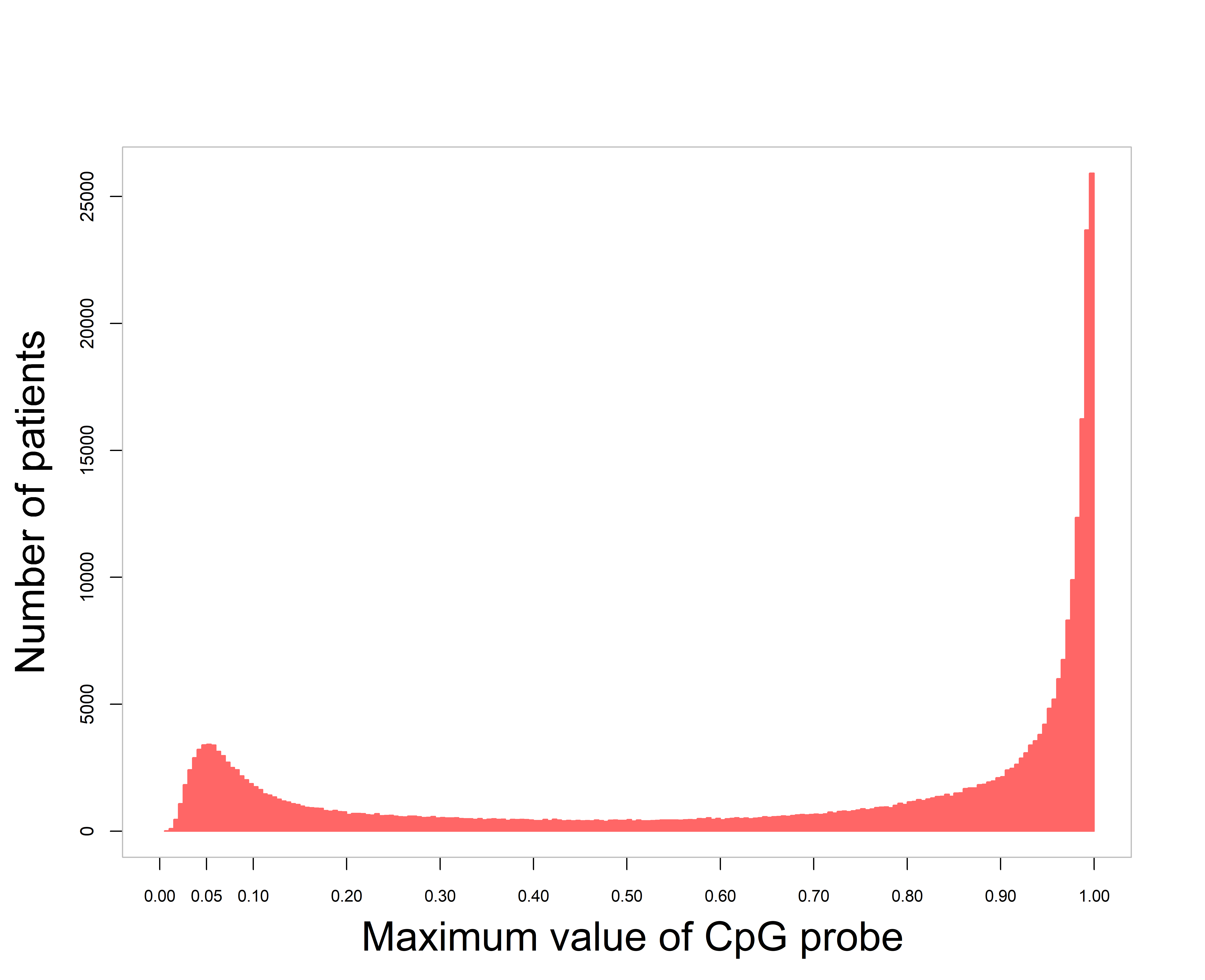

Supplement: Supplementary file 7 — Fig. S7. Distribution of maximum value of all 311891 CpG probes in non‐small‐cell lung cancer (NSCLC) patients. [file MOL2-14-2759-s007.tiff]
