# Supplementary material for: Epigenetic–smoking interaction reveals histologically heterogeneous effects of TRIM27 DNA methylation on overall survival among early‐stage NSCLC patients
Source: Mol Oncol. 2020 Sep 3;14(11):2759–74. doi: 10.1002/1878-0261.12785 (PMC7607178; doi:10.1002/1878-0261.12785)
Supplement: Supplementary file 8 — Table S1. Demographic and clinical characteristics of early‐stage NSCLC patients with gene expression data derived from TCGA. Table S2. Annotation information for 96 CpG probes located in TRIM27. Table S3. Results of association analysis of 96 DNA methylation probes of TRIM27 in LUAD samples. Table S4. Results of association analysis of 96 CpG probes of TRIM27 in LUSC samples. Table S5. Results of proportional hazards test for 96 CpG probes of TRIM27 in LUSC samples. Table S6. Comparison of smoking‐related characteristics of former and current smokers between early‐stage LUAD and LUSC. Table S7. Results of genome‐wide methylation transcription analysis of 29 genes significantly associated with cg05293407 in TCGA LUSC samples. [file MOL2-14-2759-s008.docx]

Supplementary files

**Supplementary Table S1.** Demographic and clinical characteristics of early-stage NSCLC patients with gene expression data derived from TCGA

| Characteristic | LUAD (*N* = 281) | LUSC (*N* = 277) |
| --- | --- | --- |
| Age (years) | 65.39 ± 9.58 | 67.84 ± 8.85 |
| Sex |  |  |
| Female | 145 (51.60%) | 71 (25.63%) |
| Male | 136 (48.40%) | 206 (74.37%) |
| Smoking status |  |  |
| Former | 192 (70.85%) | 87 (32.34%) |
| Current | 79 (29.15%) | 182 (67.66%) |
| Unknown | 10 | 8 |
| TNM stage |  |  |
| I | 197 (70.11) | 158 (57.04) |
| II | 84 (29.89) | 119 (42.96) |
| Chemotherapy |  |  |
| No | 95 (85.59%) | 82 (69.49%) |
| Yes | 16 (14.41%) | 36 (30.51%) |
| Unknown | 170 | 159 |
| Radiotherapy |  |  |
| No | 106 (95.50%) | 112 (94.92%) |
| Yes | 5 (4.50%) | 6 (5.08%) |
| Unknown | 170 | 159 |
| Adjuvant therapy |  |  |
| No | 90 (81.08%) | 81 (68.64%) |
| Yes | 21 (18.92%) | 37 (31.36%) |
| Unknown | 170 | 159 |
| Survival year |  |  |
| Median (95% CI) | 4.38 (3.37-4.93) | 5.08 (3.68-6.09) |
| Censored rate | 79.36% | 72.92% |

NSCLC: non-small cell lung cancer; TGCA: The Cancer Genome Atlas; LUAD: lung adenocarcinoma; LUSC: lung squamous cell carcinoma; 95% CI: 95% confidence interval

**Supplementary Table S2.** Annotation information for 96 CpG probes located in *TRIM27*

| Probe | Location | Region | CpG island | Relation to CpG islands |
| --- | --- | --- | --- | --- |
| cg00259404 | Chr6:28885568 | Body |  |  |
| cg00875200 | Chr6:28891298 | 1^st^ Exon | chr6:28890951-28892013 | Island |
| cg01521674 | Chr6:28872555 | Body |  |  |
| cg01928896 | Chr6:28872471 | Body |  |  |
| cg02593352 | Chr6:28890981 | Body | chr6:28890951-28892013 | Island |
| cg03270340 | Chr6:28891204 | 1^st^ Exon | chr6:28890951-28892013 | Island |
| cg03647327 | Chr6:28891109 | 1^st^ Exon | chr6:28890951-28892013 | Island |
| cg03671443 | Chr6:28891045 | 1^st^ Exon | chr6:28890951-28892013 | Island |
| cg05216056 | Chr6:28887836 | Body | chr6:28890951-28892013 | N_Shelf |
| **cg05293407** | **Chr6:28891967** | **TSS200** | **chr6:28890951-28892013** | **Island** |
| cg05314350 | Chr6:28874702 | Body |  |  |
| cg05594872 | Chr6:28892418 | TSS1500 | chr6:28890951-28892013 | S_Shore |
| cg05656855 | Chr6:28876300 | Body |  |  |
| cg06021189 | Chr6:28872284 | Body |  |  |
| cg06126060 | Chr6:28888883 | Body | chr6:28890951-28892013 | N_Shelf |
| cg06395692 | Chr6:28891945 | TSS200 | chr6:28890951-28892013 | Island |
| cg06600118 | Chr6:28891213 | 1^st^ Exon | chr6:28890951-28892013 | Island |
| cg07162905 | Chr6:28887928 | Body | chr6:28890951-28892013 | N_Shelf |
| cg07304943 | Chr6:28886296 | Body |  |  |
| cg07791404 | Chr6:28891716 | 5' UTR; 1^st^ Exon | chr6:28890951-28892013 | Island |
| cg08125020 | Chr6:28889357 | Body | chr6:28890951-28892013 | N_Shore |
| cg08356572 | Chr6:28891055 | 1^st^ Exon | chr6:28890951-28892013 | Island |
| cg08404201 | Chr6:28891972 | TSS1500 | chr6:28890951-28892013 | Island |
| cg08895903 | Chr6:28892077 | TSS1500 | chr6:28890951-28892013 | S_Shore |
| cg09325158 | Chr6:28891051 | 1^st^ Exon | chr6:28890951-28892013 | Island |
| cg09444060 | Chr6:28887940 | Body | chr6:28890951-28892013 | N_Shelf |
| cg09805314 | Chr6:28891289 | 1^st^ Exon | chr6:28890951-28892013 | Island |
| cg10401017 | Chr6:28887586 | Body | chr6:28890951-28892013 | N_Shelf |
| cg10718056 | Chr6:28884599 | Body |  |  |
| cg10729419 | Chr6:28872100 | Body |  |  |
| cg10905031 | Chr6:28891240 | 1^st^ Exon | chr6:28890951-28892013 | Island |
| cg11362785 | Chr6:28890979 | Body | chr6:28890951-28892013 | Island |
| cg11389756 | Chr6:28875354 | Body |  |  |
| cg11524778 | Chr6:28890618 | Body | chr6:28890951-28892013 | N_Shore |
| cg11629443 | Chr6:28891340 | 1^st^ Exon | chr6:28890951-28892013 | Island |
| cg11809702 | Chr6:28891882 | TSS200 | chr6:28890951-28892013 | Island |
| cg11886965 | Chr6:28879849 | Body |  |  |
| cg12896170 | Chr6:28890069 | Body | chr6:28890951-28892013 | N_Shore |
| cg13060704 | Chr6:28890322 | Body | chr6:28890951-28892013 | N_Shore |
| cg13110966 | Chr6:28891227 | 1^st^ Exon | chr6:28890951-28892013 | Island |
| cg13612515 | Chr6:28872335 | Body |  |  |
| cg13646645 | Chr6:28891088 | 1^st^ Exon | chr6:28890951-28892013 | Island |
| cg14059339 | Chr6:28875356 | Body |  |  |
| cg14268557 | Chr6:28874547 | Body |  |  |
| cg14633298 | Chr6:28892849 | TSS1500 | chr6:28890951-28892013 | S_Shore |
| cg14667731 | Chr6:28891990 | TSS1500 | chr6:28890951-28892013 | Island |
| cg14700841 | Chr6:28871376 | 3'UTR |  |  |
| cg14781667 | Chr6:28877495 | Body |  |  |
| cg15201399 | Chr6:28873014 | Body |  |  |
| cg15613012 | Chr6:28887742 | Body | chr6:28890951-28892013 | N_Shelf |
| cg15629064 | Chr6:28891718 | 5' UTR; 1^st^ Exon | chr6:28890951-28892013 | Island |
| cg16061301 | Chr6:28886047 | Body |  |  |
| cg16520539 | Chr6:28890872 | Body | chr6:28890951-28892013 | N_Shore |
| cg16578453 | Chr6:28891734 | 5' UTR; 1^st^ Exon | chr6:28890951-28892013 | Island |
| cg16723189 | Chr6:28890556 | Body | chr6:28890951-28892013 | N_Shore |
| cg16754082 | Chr6:28888687 | Body | chr6:28890951-28892013 | N_Shelf |
| cg16959626 | Chr6:28871583 | 3' UTR |  |  |
| cg17344091 | Chr6:28885444 | Body |  |  |
| cg17352468 | Chr6:28891388 | 1^st^ Exon | chr6:28890951-28892013 | Island |
| cg17416748 | Chr6:28872093 | Body |  |  |
| cg17564183 | Chr6:28888885 | Body | chr6:28890951-28892013 | N_Shelf |
| cg17657594 | Chr6:28892012 | TSS1500 | chr6:28890951-28892013 | Island |
| cg18104091 | Chr6:28891893 | TSS200 | chr6:28890951-28892013 | Island |
| cg18234193 | Chr6:28873520 | Body |  |  |
| cg18578954 | Chr6:28887247 | Body | chr6:28890951-28892013 | N_Shelf |
| cg18844029 | Chr6:28885017 | Body |  |  |
| cg18954047 | Chr6:28889682 | Body | chr6:28890951-28892013 | N_Shore |
| cg19276059 | Chr6:28870925 | 3' UTR |  |  |
| cg19284131 | Chr6:28889521 | Body | chr6:28890951-28892013 | N_Shore |
| cg19286631 | Chr6:28876139 | Body |  |  |
| cg19400926 | Chr6:28889996 | Body | chr6:28890951-28892013 | N_Shore |
| cg19484381 | Chr6:28890673 | Body | chr6:28890951-28892013 | N_Shore |
| cg19494464 | Chr6:28891064 | 1^st^ Exon | chr6:28890951-28892013 | Island |
| cg19519384 | Chr6:28891412 | 5' UTR; 1^st^ Exon | chr6:28890951-28892013 | Island |
| cg20370991 | Chr6:28887141 | Body | chr6:28890951-28892013 | N_Shelf |
| cg20741078 | Chr6:28891917 | TSS200 | chr6:28890951-28892013 | Island |
| cg20986887 | Chr6:28887284 | Body | chr6:28890951-28892013 | N_Shelf |
| cg21413754 | Chr6:28892079 | TSS1500 | chr6:28890951-28892013 | S_Shore |
| cg21568286 | Chr6:28891111 | 1^st^ Exon | chr6:28890951-28892013 | Island |
| cg22842085 | Chr6:28880239 | Body |  |  |
| cg22900372 | Chr6:28872654 | Body |  |  |
| cg23211158 | Chr6:28872382 | Body |  |  |
| cg23569711 | Chr6:28891915 | TSS200 | chr6:28890951-28892013 | Island |
| cg23756442 | Chr6:28892465 | TSS1500 | chr6:28890951-28892013 | S_Shore |
| cg23780580 | Chr6:28871977 | Body |  |  |
| cg24990422 | Chr6:28890682 | Body | chr6:28890951-28892013 | N_Shore |
| cg25589651 | Chr6:28891121 | 1^st^ Exon | chr6:28890951-28892013 | Island |
| cg25701364 | Chr6:28871735 | 3' UTR |  |  |
| cg25922680 | Chr6:28890911 | Body | chr6:28890951-28892013 | N_Shore |
| cg26723331 | Chr6:28872085 | Body |  |  |
| cg26892251 | Chr6:28872373 | Body |  |  |
| cg27120125 | Chr6:28892660 | TSS1500 | chr6:28890951-28892013 | S_Shore |
| cg27219662 | Chr6:28891728 | 5' UTR; 1^st^ Exon | chr6:28890951-28892013 | Island |
| cg27358207 | Chr6:28872991 | Body |  |  |
| cg27383651 | Chr6:28888809 | Body | chr6:28890951-28892013 | N_Shelf |
| cg27636813 | Chr6:28872287 | Body |  |  |

**Supplementary Table S3.** Results of association analysis of 96 DNA methylation probes of *TRIM27* in LUAD samples

| CPG probe | Discovery phase | | | | | Validation phase | | | | Combined dataset | | | |
| --- | --- | --- | --- | --- | --- | --- | --- | --- | --- | --- | --- | --- | --- |
|  | HR | 95% CI | | *P* | FDR-*q* | HR | 95% CI | | *P* | HR | 95% CI | | *P* |
| cg19286631 | 1.03 | 1.01 | 1.05 | 0.006 | 0.277 | 1.04 | 0.99 | 1.09 | 0.156 | 1.03 | 1.01 | 1.05 | 0.003 |
| cg05656855 | 1.04 | 1.01 | 1.07 | 0.004 | 0.277 | 1.00 | 0.96 | 1.05 | 0.842 | 1.03 | 1.01 | 1.05 | 0.009 |
| cg23569711 | 1.23 | 1.04 | 1.45 | 0.016 | 0.521 | 0.98 | 0.73 | 1.32 | 0.892 | 1.16 | 1.01 | 1.34 | 0.040 |
| cg11629443 | 1.15 | 0.97 | 1.37 | 0.115 | 0.697 | 1.39 | 0.99 | 1.96 | 0.055 | 1.18 | 1.01 | 1.38 | 0.031 |
| cg19494464 | 1.14 | 0.97 | 1.33 | 0.110 | 0.697 | 1.28 | 0.95 | 1.72 | 0.099 | 1.16 | 1.01 | 1.33 | 0.041 |
| cg22842085 | 1.01 | 1.00 | 1.03 | 0.116 | 0.697 | 1.10 | 0.97 | 1.23 | 0.132 | 1.02 | 1.00 | 1.03 | 0.079 |
| cg01928896 | 1.07 | 0.99 | 1.14 | 0.070 | 0.697 | 1.10 | 0.94 | 1.29 | 0.242 | 1.06 | 1.00 | 1.13 | 0.063 |
| cg14633298 | 1.02 | 1.00 | 1.04 | 0.064 | 0.697 | 0.95 | 0.88 | 1.04 | 0.278 | 1.01 | 1.00 | 1.03 | 0.090 |
| cg16520539 | 1.17 | 0.97 | 1.42 | 0.104 | 0.697 | 1.26 | 0.82 | 1.93 | 0.287 | 1.19 | 1.00 | 1.42 | 0.044 |
| cg27636813 | 0.90 | 0.79 | 1.02 | 0.094 | 0.697 | 1.11 | 0.91 | 1.36 | 0.293 | 0.94 | 0.85 | 1.05 | 0.277 |
| cg27120125 | 1.01 | 1.00 | 1.02 | 0.112 | 0.697 | 1.01 | 0.99 | 1.03 | 0.465 | 1.01 | 1.00 | 1.02 | 0.042 |
| cg09444060 | 1.03 | 1.00 | 1.06 | 0.097 | 0.697 | 1.06 | 0.85 | 1.32 | 0.586 | 1.03 | 1.00 | 1.06 | 0.080 |
| cg11809702 | 0.79 | 0.59 | 1.05 | 0.101 | 0.697 | 0.87 | 0.51 | 1.50 | 0.622 | 0.83 | 0.65 | 1.06 | 0.134 |
| cg27383651 | 1.01 | 1.00 | 1.03 | 0.090 | 0.697 | 1.00 | 0.98 | 1.03 | 0.821 | 1.01 | 1.00 | 1.02 | 0.120 |
| cg00875200 | 0.85 | 0.71 | 1.01 | 0.063 | 0.697 | 1.02 | 0.63 | 1.65 | 0.948 | 0.87 | 0.74 | 1.03 | 0.099 |
| cg16754082 | 1.06 | 0.98 | 1.15 | 0.116 | 0.697 | 1.00 | 0.77 | 1.29 | 0.993 | 1.05 | 0.98 | 1.13 | 0.154 |
| cg27358207 | 1.01 | 0.99 | 1.04 | 0.151 | 0.707 | 0.90 | 0.81 | 1.00 | 0.042 | 1.01 | 0.99 | 1.03 | 0.247 |
| cg26723331 | 1.13 | 0.95 | 1.33 | 0.162 | 0.707 | 1.51 | 1.01 | 2.25 | 0.045 | 1.17 | 1.00 | 1.37 | 0.048 |
| cg21568286 | 1.17 | 0.95 | 1.44 | 0.132 | 0.707 | 1.43 | 0.90 | 2.27 | 0.127 | 1.18 | 0.98 | 1.42 | 0.081 |
| cg05594872 | 0.99 | 0.98 | 1.00 | 0.140 | 0.707 | 1.04 | 0.99 | 1.10 | 0.156 | 0.99 | 0.98 | 1.01 | 0.312 |
| cg03270340 | 1.03 | 0.99 | 1.06 | 0.147 | 0.707 | 0.92 | 0.81 | 1.05 | 0.223 | 1.02 | 0.98 | 1.05 | 0.343 |
| cg07791404 | 0.91 | 0.80 | 1.04 | 0.162 | 0.707 | 0.96 | 0.75 | 1.24 | 0.783 | 0.92 | 0.82 | 1.03 | 0.138 |
| cg10718056 | 0.99 | 0.98 | 1.01 | 0.279 | 0.826 | 0.97 | 0.94 | 1.00 | 0.076 | 0.99 | 0.98 | 1.00 | 0.084 |
| cg14781667 | 1.02 | 0.98 | 1.06 | 0.267 | 0.826 | 0.90 | 0.78 | 1.04 | 0.172 | 1.01 | 0.98 | 1.05 | 0.446 |
| cg13646645 | 0.87 | 0.70 | 1.08 | 0.200 | 0.826 | 1.23 | 0.85 | 1.79 | 0.279 | 0.93 | 0.76 | 1.13 | 0.446 |
| cg16723189 | 1.02 | 0.98 | 1.07 | 0.290 | 0.826 | 1.11 | 0.88 | 1.39 | 0.389 | 1.02 | 0.98 | 1.06 | 0.293 |
| cg25701364 | 0.97 | 0.92 | 1.02 | 0.254 | 0.826 | 1.05 | 0.94 | 1.16 | 0.393 | 0.98 | 0.94 | 1.03 | 0.471 |
| cg17564183 | 1.02 | 0.98 | 1.07 | 0.271 | 0.826 | 1.03 | 0.95 | 1.12 | 0.463 | 1.02 | 0.99 | 1.06 | 0.230 |
| cg07304943 | 1.06 | 0.96 | 1.16 | 0.236 | 0.826 | 1.08 | 0.88 | 1.33 | 0.472 | 1.05 | 0.97 | 1.14 | 0.243 |
| cg14667731 | 1.11 | 0.91 | 1.37 | 0.293 | 0.826 | 1.08 | 0.76 | 1.55 | 0.660 | 1.10 | 0.92 | 1.31 | 0.294 |
| cg06126060 | 1.03 | 0.98 | 1.08 | 0.230 | 0.826 | 0.96 | 0.79 | 1.17 | 0.664 | 1.02 | 0.98 | 1.07 | 0.281 |
| cg08404201 | 1.09 | 0.95 | 1.24 | 0.212 | 0.826 | 1.06 | 0.79 | 1.43 | 0.680 | 1.07 | 0.95 | 1.21 | 0.248 |
| cg22900372 | 1.04 | 0.97 | 1.11 | 0.266 | 0.826 | 0.98 | 0.78 | 1.24 | 0.894 | 1.03 | 0.97 | 1.10 | 0.278 |
| cg17416748 | 1.06 | 0.97 | 1.16 | 0.232 | 0.826 | 0.99 | 0.85 | 1.16 | 0.925 | 1.04 | 0.96 | 1.12 | 0.374 |
| cg03647327 | 1.18 | 0.83 | 1.67 | 0.364 | 0.833 | 2.34 | 0.93 | 5.87 | 0.070 | 1.28 | 0.93 | 1.77 | 0.130 |
| cg19284131 | 1.01 | 0.99 | 1.04 | 0.318 | 0.833 | 0.96 | 0.90 | 1.03 | 0.271 | 1.01 | 0.98 | 1.03 | 0.617 |
| cg24990422 | 1.09 | 0.91 | 1.31 | 0.359 | 0.833 | 1.16 | 0.85 | 1.59 | 0.344 | 1.12 | 0.95 | 1.30 | 0.172 |
| cg23211158 | 1.05 | 0.95 | 1.15 | 0.333 | 0.833 | 1.09 | 0.90 | 1.32 | 0.381 | 1.05 | 0.96 | 1.14 | 0.283 |
| cg13060704 | 0.99 | 0.98 | 1.01 | 0.368 | 0.833 | 1.01 | 0.98 | 1.04 | 0.428 | 1.00 | 0.99 | 1.01 | 0.679 |
| cg00259404 | 0.99 | 0.98 | 1.01 | 0.375 | 0.833 | 0.99 | 0.97 | 1.02 | 0.460 | 0.99 | 0.98 | 1.00 | 0.211 |
| cg18844029 | 1.03 | 0.96 | 1.11 | 0.382 | 0.833 | 0.97 | 0.83 | 1.12 | 0.642 | 1.02 | 0.96 | 1.09 | 0.552 |
| cg11362785 | 0.92 | 0.78 | 1.10 | 0.372 | 0.833 | 0.96 | 0.66 | 1.40 | 0.831 | 0.93 | 0.79 | 1.09 | 0.353 |
| cg13612515 | 1.03 | 0.97 | 1.10 | 0.353 | 0.833 | 1.01 | 0.92 | 1.11 | 0.877 | 1.02 | 0.97 | 1.08 | 0.362 |
| cg10905031 | 1.03 | 0.97 | 1.08 | 0.381 | 0.833 | 1.00 | 0.85 | 1.19 | 0.989 | 1.02 | 0.97 | 1.08 | 0.415 |
| cg09325158 | 1.05 | 0.94 | 1.16 | 0.424 | 0.851 | 0.91 | 0.76 | 1.08 | 0.266 | 1.01 | 0.93 | 1.11 | 0.765 |
| cg18234193 | 0.98 | 0.91 | 1.04 | 0.465 | 0.851 | 1.08 | 0.93 | 1.24 | 0.319 | 0.99 | 0.93 | 1.05 | 0.761 |
| cg16061301 | 1.03 | 0.95 | 1.12 | 0.470 | 0.851 | 1.05 | 0.91 | 1.21 | 0.511 | 1.03 | 0.96 | 1.10 | 0.430 |
| cg14700841 | 1.02 | 0.97 | 1.09 | 0.414 | 0.851 | 1.06 | 0.86 | 1.31 | 0.563 | 1.03 | 0.97 | 1.09 | 0.366 |
| cg10729419 | 1.04 | 0.94 | 1.15 | 0.432 | 0.851 | 1.04 | 0.88 | 1.24 | 0.654 | 1.03 | 0.95 | 1.12 | 0.444 |
| cg18104091 | 0.90 | 0.70 | 1.17 | 0.435 | 0.851 | 0.90 | 0.57 | 1.43 | 0.666 | 0.91 | 0.73 | 1.13 | 0.391 |
| cg19400926 | 0.99 | 0.98 | 1.01 | 0.437 | 0.851 | 0.99 | 0.96 | 1.03 | 0.680 | 0.99 | 0.98 | 1.01 | 0.428 |
| cg16578453 | 0.90 | 0.68 | 1.18 | 0.446 | 0.851 | 1.09 | 0.61 | 1.97 | 0.769 | 0.95 | 0.75 | 1.22 | 0.696 |
| cg14268557 | 1.01 | 0.99 | 1.02 | 0.459 | 0.851 | 1.00 | 0.96 | 1.03 | 0.901 | 1.00 | 0.99 | 1.02 | 0.584 |
| cg05216056 | 0.99 | 0.97 | 1.02 | 0.494 | 0.858 | 0.95 | 0.90 | 1.00 | 0.066 | 0.98 | 0.96 | 1.01 | 0.138 |
| cg02593352 | 0.88 | 0.60 | 1.28 | 0.500 | 0.858 | 0.65 | 0.28 | 1.50 | 0.311 | 0.84 | 0.60 | 1.19 | 0.328 |
| cg19276059 | 0.98 | 0.94 | 1.03 | 0.486 | 0.858 | 1.05 | 0.93 | 1.19 | 0.433 | 0.99 | 0.95 | 1.03 | 0.655 |
| cg16959626 | 0.97 | 0.90 | 1.06 | 0.530 | 0.876 | 1.23 | 1.00 | 1.52 | 0.046 | 1.01 | 0.94 | 1.09 | 0.769 |
| cg03671443 | 0.97 | 0.88 | 1.07 | 0.551 | 0.876 | 0.86 | 0.71 | 1.04 | 0.122 | 0.95 | 0.87 | 1.03 | 0.226 |
| cg06021189 | 0.98 | 0.93 | 1.04 | 0.543 | 0.876 | 1.02 | 0.93 | 1.12 | 0.657 | 0.99 | 0.95 | 1.04 | 0.717 |
| cg17344091 | 1.00 | 0.99 | 1.01 | 0.554 | 0.876 | 1.00 | 0.98 | 1.02 | 0.934 | 1.00 | 0.99 | 1.01 | 0.720 |
| cg01521674 | 1.03 | 0.94 | 1.12 | 0.557 | 0.876 | 1.01 | 0.82 | 1.24 | 0.952 | 1.02 | 0.94 | 1.10 | 0.691 |
| cg12896170 | 1.00 | 0.99 | 1.01 | 0.581 | 0.898 | 1.01 | 0.99 | 1.03 | 0.368 | 1.00 | 0.99 | 1.01 | 0.975 |
| cg20986887 | 1.00 | 0.99 | 1.01 | 0.589 | 0.898 | 1.00 | 0.98 | 1.01 | 0.683 | 1.00 | 0.99 | 1.00 | 0.447 |
| cg20741078 | 1.07 | 0.82 | 1.39 | 0.634 | 0.922 | 0.64 | 0.39 | 1.04 | 0.073 | 0.94 | 0.76 | 1.17 | 0.597 |
| cg23756442 | 1.00 | 0.99 | 1.01 | 0.627 | 0.922 | 1.00 | 0.97 | 1.02 | 0.790 | 1.00 | 0.99 | 1.01 | 0.734 |
| cg26892251 | 0.96 | 0.83 | 1.12 | 0.620 | 0.922 | 1.02 | 0.82 | 1.26 | 0.871 | 0.96 | 0.85 | 1.08 | 0.501 |
| cg15201399 | 1.01 | 0.96 | 1.06 | 0.651 | 0.930 | 0.84 | 0.68 | 1.04 | 0.108 | 1.00 | 0.96 | 1.05 | 0.868 |
| cg21413754 | 0.96 | 0.81 | 1.14 | 0.676 | 0.930 | 0.79 | 0.49 | 1.26 | 0.321 | 0.93 | 0.78 | 1.10 | 0.392 |
| cg06395692 | 1.07 | 0.77 | 1.49 | 0.678 | 0.930 | 1.25 | 0.73 | 2.13 | 0.419 | 1.08 | 0.82 | 1.41 | 0.595 |
| cg25922680 | 1.04 | 0.88 | 1.22 | 0.668 | 0.930 | 1.08 | 0.76 | 1.54 | 0.676 | 1.04 | 0.89 | 1.20 | 0.638 |
| cg23780580 | 1.02 | 0.91 | 1.14 | 0.697 | 0.936 | 1.16 | 0.94 | 1.42 | 0.162 | 1.06 | 0.96 | 1.16 | 0.274 |
| cg08356572 | 1.02 | 0.93 | 1.11 | 0.712 | 0.936 | 0.91 | 0.76 | 1.10 | 0.331 | 1.00 | 0.93 | 1.09 | 0.926 |
| cg10401017 | 1.00 | 0.99 | 1.02 | 0.722 | 0.936 | 1.00 | 0.97 | 1.04 | 0.903 | 1.00 | 0.99 | 1.01 | 0.847 |
| cg15613012 | 1.01 | 0.95 | 1.07 | 0.713 | 0.936 | 0.99 | 0.84 | 1.17 | 0.936 | 1.00 | 0.95 | 1.06 | 0.884 |
| cg19519384 | 1.02 | 0.89 | 1.17 | 0.742 | 0.949 | 0.84 | 0.62 | 1.13 | 0.248 | 0.99 | 0.88 | 1.13 | 0.913 |
| cg11886965 | 1.00 | 0.96 | 1.03 | 0.774 | 0.961 | 0.97 | 0.92 | 1.03 | 0.336 | 0.99 | 0.96 | 1.01 | 0.330 |
| cg09805314 | 1.02 | 0.90 | 1.15 | 0.781 | 0.961 | 0.73 | 0.38 | 1.42 | 0.354 | 1.01 | 0.89 | 1.14 | 0.868 |
| cg11524778 | 0.97 | 0.81 | 1.17 | 0.767 | 0.961 | 1.17 | 0.83 | 1.63 | 0.371 | 1.02 | 0.87 | 1.19 | 0.836 |
| cg17352468 | 1.01 | 0.92 | 1.12 | 0.800 | 0.972 | 1.27 | 1.01 | 1.59 | 0.037 | 1.05 | 0.96 | 1.14 | 0.317 |
| cg13110966 | 0.98 | 0.85 | 1.14 | 0.831 | 0.997 | 0.82 | 0.56 | 1.19 | 0.294 | 0.95 | 0.83 | 1.08 | 0.431 |
| cg05293407 | 0.98 | 0.77 | 1.25 | 0.893 | 0.998 | 1.38 | 0.92 | 2.07 | 0.115 | 1.08 | 0.87 | 1.33 | 0.493 |
| cg17657594 | 1.00 | 0.90 | 1.11 | 0.957 | 0.998 | 1.43 | 0.87 | 2.35 | 0.160 | 1.01 | 0.91 | 1.12 | 0.810 |
| cg25589651 | 1.00 | 0.93 | 1.07 | 0.910 | 0.998 | 0.90 | 0.77 | 1.04 | 0.162 | 0.97 | 0.91 | 1.04 | 0.362 |
| cg18578954 | 1.00 | 0.99 | 1.01 | 0.888 | 0.998 | 0.99 | 0.97 | 1.01 | 0.254 | 1.00 | 0.99 | 1.00 | 0.528 |
| cg08895903 | 1.00 | 0.84 | 1.19 | 0.998 | 0.998 | 1.17 | 0.84 | 1.62 | 0.362 | 1.03 | 0.88 | 1.20 | 0.708 |
| cg18954047 | 1.00 | 0.99 | 1.01 | 0.969 | 0.998 | 1.01 | 0.99 | 1.02 | 0.441 | 1.00 | 0.99 | 1.01 | 0.686 |
| cg20370991 | 1.00 | 0.98 | 1.02 | 0.935 | 0.998 | 0.98 | 0.90 | 1.06 | 0.571 | 1.00 | 0.98 | 1.02 | 0.811 |
| cg19484381 | 1.00 | 0.96 | 1.03 | 0.897 | 0.998 | 0.98 | 0.92 | 1.04 | 0.580 | 0.99 | 0.97 | 1.03 | 0.730 |
| cg05314350 | 1.00 | 0.98 | 1.01 | 0.877 | 0.998 | 0.99 | 0.96 | 1.02 | 0.592 | 1.00 | 0.98 | 1.01 | 0.596 |
| cg14059339 | 1.00 | 0.99 | 1.01 | 0.950 | 0.998 | 1.00 | 0.99 | 1.02 | 0.645 | 1.00 | 0.99 | 1.01 | 0.952 |
| cg15629064 | 0.99 | 0.84 | 1.18 | 0.948 | 0.998 | 1.08 | 0.76 | 1.53 | 0.682 | 1.01 | 0.87 | 1.18 | 0.858 |
| cg08125020 | 1.00 | 0.93 | 1.07 | 0.898 | 0.998 | 0.97 | 0.83 | 1.13 | 0.700 | 0.99 | 0.93 | 1.05 | 0.700 |
| cg11389756 | 1.00 | 0.99 | 1.01 | 0.995 | 0.998 | 1.00 | 0.99 | 1.02 | 0.829 | 1.00 | 0.99 | 1.01 | 0.862 |
| cg27219662 | 1.01 | 0.89 | 1.14 | 0.908 | 0.998 | 1.03 | 0.78 | 1.36 | 0.831 | 1.00 | 0.89 | 1.12 | 0.988 |
| cg07162905 | 1.00 | 0.87 | 1.15 | 0.983 | 0.998 | 0.98 | 0.72 | 1.33 | 0.888 | 0.99 | 0.87 | 1.12 | 0.837 |
| cg06600118 | 1.00 | 0.80 | 1.24 | 0.967 | 0.998 | 1.02 | 0.66 | 1.56 | 0.939 | 1.02 | 0.84 | 1.24 | 0.853 |

Hazard ratio (HR), 95% confidence interval (95% CI), and *P*-value were derived from Cox proportional hazards model adjusted for age, sex, clinical stage, smoking status, and study centre.

FDR: false discovery rate

**Supplementary Table S4.** Results of association analysis of 96 CpG probes of *TRIM27* in LUSC samples

| CPG probe | Discovery phase | | | | | Validation phase | | | | Combined dataset | | | |
| --- | --- | --- | --- | --- | --- | --- | --- | --- | --- | --- | --- | --- | --- |
|  | HR | 95% CI | | *P* | FDR-*q* | HR | 95% CI | | *P* | HR | 95% CI | | *P* |
| **cg05293407** | **2.10** | **1.41** | **3.12** | **2.70E-04** | **0.026** | **1.49** | **1.07** | **2.07** | **0.018** | **1.65** | **1.30** | **2.09** | **4.52E-05** |
| cg09325158 | 1.33 | 1.13 | 1.57 | 0.001 | 0.029 | 1.04 | 0.89 | 1.20 | 0.654 | 1.15 | 1.03 | 1.27 | 0.012 |
| cg19484381 | 1.10 | 1.03 | 1.18 | 0.007 | 0.237 | 1.06 | 0.99 | 1.13 | 0.111 | 1.07 | 1.02 | 1.13 | 0.004 |
| cg25589651 | 1.17 | 1.03 | 1.32 | 0.016 | 0.344 | 1.05 | 0.94 | 1.17 | 0.408 | 1.10 | 1.01 | 1.19 | 0.021 |
| cg06600118 | 1.54 | 1.08 | 2.20 | 0.018 | 0.344 | 1.09 | 0.65 | 1.83 | 0.737 | 1.30 | 0.97 | 1.75 | 0.084 |
| cg08356572 | 1.19 | 0.99 | 1.43 | 0.069 | 0.774 | 1.16 | 0.98 | 1.37 | 0.078 | 1.18 | 1.05 | 1.33 | 0.006 |
| cg08125020 | 0.83 | 0.68 | 1.01 | 0.059 | 0.774 | 1.20 | 0.95 | 1.51 | 0.128 | 0.97 | 0.85 | 1.11 | 0.680 |
| cg06395692 | 1.70 | 0.98 | 2.96 | 0.061 | 0.774 | 1.18 | 0.73 | 1.89 | 0.503 | 1.30 | 0.92 | 1.85 | 0.136 |
| cg17352468 | 1.23 | 0.98 | 1.54 | 0.073 | 0.774 | 0.98 | 0.80 | 1.20 | 0.853 | 1.08 | 0.94 | 1.25 | 0.284 |
| cg03270340 | 1.08 | 0.98 | 1.19 | 0.105 | 0.790 | 1.12 | 1.00 | 1.25 | 0.050 | 1.09 | 1.02 | 1.17 | 0.016 |
| cg18954047 | 0.99 | 0.97 | 1.00 | 0.083 | 0.790 | 1.01 | 0.99 | 1.02 | 0.470 | 1.00 | 0.99 | 1.01 | 0.635 |
| cg05656855 | 0.95 | 0.89 | 1.01 | 0.107 | 0.790 | 1.02 | 0.95 | 1.10 | 0.515 | 0.99 | 0.94 | 1.03 | 0.584 |
| cg07791404 | 1.18 | 0.97 | 1.44 | 0.101 | 0.790 | 1.06 | 0.87 | 1.29 | 0.560 | 1.11 | 0.97 | 1.28 | 0.133 |
| cg23756442 | 1.02 | 0.99 | 1.04 | 0.131 | 0.872 | 1.02 | 1.00 | 1.04 | 0.021 | 1.02 | 1.00 | 1.03 | 0.011 |
| cg15629064 | 1.19 | 0.95 | 1.49 | 0.136 | 0.872 | 1.05 | 0.86 | 1.27 | 0.640 | 1.11 | 0.97 | 1.28 | 0.137 |
| cg23569711 | 1.22 | 0.89 | 1.67 | 0.209 | 0.893 | 1.41 | 1.09 | 1.82 | 0.009 | 1.28 | 1.06 | 1.55 | 0.011 |
| cg19284131 | 0.96 | 0.88 | 1.05 | 0.326 | 0.893 | 1.09 | 1.00 | 1.19 | 0.042 | 1.02 | 0.97 | 1.08 | 0.434 |
| cg16959626 | 1.13 | 0.91 | 1.41 | 0.258 | 0.893 | 1.16 | 0.98 | 1.38 | 0.091 | 1.12 | 0.98 | 1.27 | 0.088 |
| cg10729419 | 0.92 | 0.80 | 1.07 | 0.273 | 0.893 | 1.13 | 0.97 | 1.31 | 0.112 | 1.01 | 0.91 | 1.12 | 0.845 |
| cg18578954 | 0.99 | 0.98 | 1.01 | 0.236 | 0.893 | 0.99 | 0.98 | 1.00 | 0.114 | 0.99 | 0.98 | 1.00 | 0.115 |
| cg16520539 | 1.28 | 0.87 | 1.88 | 0.216 | 0.893 | 1.29 | 0.91 | 1.83 | 0.148 | 1.25 | 0.97 | 1.61 | 0.082 |
| cg11629443 | 1.26 | 0.85 | 1.86 | 0.245 | 0.893 | 1.21 | 0.89 | 1.64 | 0.225 | 1.23 | 0.97 | 1.55 | 0.086 |
| cg20986887 | 0.99 | 0.97 | 1.01 | 0.218 | 0.893 | 0.99 | 0.98 | 1.01 | 0.261 | 0.99 | 0.98 | 1.00 | 0.241 |
| cg22842085 | 0.98 | 0.95 | 1.02 | 0.322 | 0.893 | 1.05 | 0.96 | 1.14 | 0.279 | 0.99 | 0.96 | 1.03 | 0.751 |
| cg02593352 | 0.65 | 0.32 | 1.30 | 0.221 | 0.893 | 0.70 | 0.35 | 1.41 | 0.322 | 0.63 | 0.39 | 1.03 | 0.068 |
| cg11362785 | 0.85 | 0.66 | 1.10 | 0.225 | 0.893 | 1.12 | 0.80 | 1.57 | 0.510 | 0.92 | 0.77 | 1.09 | 0.327 |
| cg20741078 | 1.19 | 0.86 | 1.64 | 0.292 | 0.893 | 1.15 | 0.74 | 1.78 | 0.538 | 1.13 | 0.88 | 1.44 | 0.337 |
| cg15613012 | 0.87 | 0.70 | 1.09 | 0.224 | 0.893 | 0.96 | 0.82 | 1.13 | 0.599 | 0.93 | 0.82 | 1.05 | 0.221 |
| cg25701364 | 0.96 | 0.88 | 1.04 | 0.298 | 0.893 | 0.98 | 0.89 | 1.08 | 0.642 | 0.97 | 0.91 | 1.03 | 0.333 |
| cg16061301 | 0.87 | 0.65 | 1.16 | 0.335 | 0.893 | 1.05 | 0.82 | 1.33 | 0.704 | 0.95 | 0.79 | 1.13 | 0.554 |
| cg27383651 | 0.98 | 0.95 | 1.01 | 0.222 | 0.893 | 0.99 | 0.97 | 1.02 | 0.710 | 0.99 | 0.97 | 1.01 | 0.162 |
| cg26723331 | 0.78 | 0.55 | 1.11 | 0.170 | 0.893 | 1.05 | 0.77 | 1.45 | 0.744 | 0.92 | 0.73 | 1.16 | 0.480 |
| cg14700841 | 0.89 | 0.71 | 1.11 | 0.309 | 0.893 | 0.98 | 0.80 | 1.20 | 0.874 | 0.93 | 0.81 | 1.07 | 0.326 |
| cg14781667 | 0.94 | 0.83 | 1.06 | 0.317 | 0.893 | 1.01 | 0.85 | 1.19 | 0.921 | 0.98 | 0.89 | 1.07 | 0.658 |
| cg00875200 | 1.18 | 0.92 | 1.51 | 0.200 | 0.893 | 1.01 | 0.71 | 1.44 | 0.953 | 1.13 | 0.93 | 1.38 | 0.223 |
| cg11886965 | 0.97 | 0.91 | 1.03 | 0.335 | 0.893 | 1.00 | 0.93 | 1.07 | 0.964 | 0.98 | 0.94 | 1.03 | 0.399 |
| cg13060704 | 1.00 | 0.97 | 1.02 | 0.726 | 0.897 | 1.04 | 1.01 | 1.06 | 0.010 | 1.01 | 1.00 | 1.03 | 0.140 |
| cg23211158 | 0.91 | 0.75 | 1.11 | 0.357 | 0.897 | 1.28 | 1.05 | 1.56 | 0.014 | 1.06 | 0.93 | 1.22 | 0.385 |
| cg25922680 | 0.93 | 0.70 | 1.24 | 0.639 | 0.897 | 1.40 | 1.02 | 1.93 | 0.036 | 1.05 | 0.87 | 1.27 | 0.603 |
| cg27120125 | 1.01 | 0.99 | 1.03 | 0.403 | 0.897 | 1.02 | 1.00 | 1.04 | 0.056 | 1.01 | 1.00 | 1.03 | 0.103 |
| cg05314350 | 0.99 | 0.96 | 1.02 | 0.502 | 0.897 | 0.97 | 0.94 | 1.00 | 0.069 | 0.98 | 0.96 | 1.01 | 0.145 |
| cg10718056 | 0.99 | 0.96 | 1.02 | 0.572 | 0.897 | 0.97 | 0.94 | 1.00 | 0.070 | 0.99 | 0.97 | 1.01 | 0.201 |
| cg21413754 | 1.07 | 0.63 | 1.81 | 0.800 | 0.897 | 0.60 | 0.33 | 1.07 | 0.084 | 0.80 | 0.54 | 1.18 | 0.259 |
| cg00259404 | 0.99 | 0.97 | 1.01 | 0.367 | 0.897 | 0.98 | 0.96 | 1.00 | 0.090 | 0.99 | 0.97 | 1.00 | 0.083 |
| cg10401017 | 0.99 | 0.95 | 1.04 | 0.698 | 0.897 | 0.96 | 0.91 | 1.01 | 0.108 | 0.98 | 0.94 | 1.01 | 0.220 |
| cg19519384 | 1.08 | 0.89 | 1.31 | 0.432 | 0.897 | 0.81 | 0.62 | 1.06 | 0.123 | 0.97 | 0.83 | 1.13 | 0.652 |
| cg18104091 | 1.07 | 0.78 | 1.47 | 0.662 | 0.897 | 1.43 | 0.91 | 2.26 | 0.123 | 1.16 | 0.89 | 1.50 | 0.265 |
| cg17344091 | 1.00 | 0.98 | 1.02 | 0.707 | 0.897 | 0.99 | 0.97 | 1.00 | 0.138 | 0.99 | 0.98 | 1.01 | 0.381 |
| cg21568286 | 1.11 | 0.70 | 1.77 | 0.650 | 0.897 | 1.32 | 0.88 | 1.97 | 0.177 | 1.26 | 0.94 | 1.69 | 0.126 |
| cg18234193 | 0.96 | 0.84 | 1.09 | 0.496 | 0.897 | 1.07 | 0.97 | 1.17 | 0.196 | 1.02 | 0.95 | 1.09 | 0.624 |
| cg26892251 | 0.90 | 0.68 | 1.18 | 0.450 | 0.897 | 1.20 | 0.91 | 1.60 | 0.199 | 1.04 | 0.86 | 1.27 | 0.669 |
| cg01928896 | 1.03 | 0.91 | 1.17 | 0.633 | 0.897 | 0.93 | 0.83 | 1.04 | 0.210 | 0.96 | 0.89 | 1.04 | 0.366 |
| cg16578453 | 1.16 | 0.67 | 2.00 | 0.595 | 0.897 | 1.42 | 0.82 | 2.45 | 0.212 | 1.25 | 0.86 | 1.82 | 0.235 |
| cg06021189 | 0.97 | 0.88 | 1.07 | 0.523 | 0.897 | 1.06 | 0.97 | 1.17 | 0.212 | 1.01 | 0.95 | 1.08 | 0.692 |
| cg22900372 | 1.07 | 0.90 | 1.27 | 0.476 | 0.897 | 1.18 | 0.91 | 1.53 | 0.214 | 1.12 | 0.98 | 1.29 | 0.102 |
| cg03647327 | 1.18 | 0.53 | 2.59 | 0.688 | 0.897 | 1.73 | 0.71 | 4.22 | 0.228 | 1.31 | 0.72 | 2.36 | 0.376 |
| cg09444060 | 0.96 | 0.84 | 1.09 | 0.506 | 0.897 | 1.12 | 0.93 | 1.35 | 0.230 | 1.00 | 0.90 | 1.12 | 0.938 |
| cg17564183 | 0.99 | 0.93 | 1.05 | 0.738 | 0.897 | 1.05 | 0.97 | 1.14 | 0.232 | 1.00 | 0.95 | 1.05 | 0.947 |
| cg10905031 | 1.04 | 0.89 | 1.20 | 0.641 | 0.897 | 1.09 | 0.92 | 1.29 | 0.301 | 1.06 | 0.95 | 1.18 | 0.316 |
| cg12896170 | 0.99 | 0.98 | 1.01 | 0.567 | 0.897 | 1.01 | 0.99 | 1.02 | 0.323 | 1.00 | 0.99 | 1.01 | 0.769 |
| cg05216056 | 0.99 | 0.94 | 1.04 | 0.601 | 0.897 | 0.97 | 0.92 | 1.03 | 0.339 | 0.98 | 0.95 | 1.02 | 0.353 |
| cg14268557 | 0.99 | 0.95 | 1.03 | 0.480 | 0.897 | 0.99 | 0.96 | 1.02 | 0.347 | 0.99 | 0.96 | 1.01 | 0.213 |
| cg11389756 | 1.00 | 0.99 | 1.02 | 0.576 | 0.897 | 0.99 | 0.98 | 1.01 | 0.403 | 1.00 | 0.99 | 1.01 | 0.939 |
| cg27358207 | 1.01 | 0.95 | 1.08 | 0.750 | 0.897 | 1.07 | 0.90 | 1.26 | 0.449 | 1.02 | 0.96 | 1.09 | 0.458 |
| cg14667731 | 0.95 | 0.66 | 1.37 | 0.789 | 0.897 | 1.16 | 0.77 | 1.74 | 0.478 | 1.00 | 0.76 | 1.31 | 0.994 |
| cg19400926 | 1.01 | 0.98 | 1.04 | 0.639 | 0.897 | 1.01 | 0.98 | 1.04 | 0.508 | 1.01 | 0.99 | 1.03 | 0.554 |
| cg23780580 | 0.93 | 0.75 | 1.15 | 0.504 | 0.897 | 1.06 | 0.88 | 1.28 | 0.529 | 1.00 | 0.87 | 1.16 | 0.945 |
| cg08895903 | 1.19 | 0.77 | 1.85 | 0.432 | 0.897 | 0.92 | 0.68 | 1.25 | 0.590 | 1.01 | 0.79 | 1.30 | 0.912 |
| cg16723189 | 1.04 | 0.84 | 1.28 | 0.743 | 0.897 | 0.95 | 0.77 | 1.16 | 0.608 | 0.98 | 0.85 | 1.13 | 0.822 |
| cg16754082 | 1.02 | 0.86 | 1.21 | 0.813 | 0.897 | 0.94 | 0.75 | 1.18 | 0.614 | 1.00 | 0.87 | 1.13 | 0.948 |
| cg06126060 | 0.93 | 0.80 | 1.09 | 0.383 | 0.897 | 1.03 | 0.89 | 1.19 | 0.672 | 0.97 | 0.87 | 1.07 | 0.519 |
| cg15201399 | 1.10 | 0.89 | 1.36 | 0.382 | 0.897 | 1.05 | 0.83 | 1.32 | 0.701 | 1.10 | 0.95 | 1.28 | 0.187 |
| cg18844029 | 0.96 | 0.86 | 1.08 | 0.519 | 0.897 | 0.97 | 0.84 | 1.13 | 0.712 | 0.99 | 0.90 | 1.08 | 0.814 |
| cg13612515 | 1.01 | 0.93 | 1.11 | 0.766 | 0.897 | 1.01 | 0.94 | 1.09 | 0.767 | 1.01 | 0.95 | 1.06 | 0.849 |
| cg14633298 | 1.01 | 0.97 | 1.05 | 0.638 | 0.897 | 1.01 | 0.96 | 1.05 | 0.781 | 1.01 | 0.98 | 1.04 | 0.538 |
| cg08404201 | 1.06 | 0.85 | 1.32 | 0.634 | 0.897 | 0.97 | 0.76 | 1.24 | 0.801 | 1.04 | 0.88 | 1.22 | 0.668 |
| cg03671443 | 1.05 | 0.91 | 1.21 | 0.513 | 0.897 | 1.02 | 0.86 | 1.21 | 0.804 | 1.02 | 0.92 | 1.14 | 0.691 |
| cg11809702 | 1.06 | 0.75 | 1.52 | 0.731 | 0.897 | 0.95 | 0.65 | 1.41 | 0.807 | 1.02 | 0.78 | 1.32 | 0.894 |
| cg20370991 | 1.04 | 0.83 | 1.30 | 0.727 | 0.897 | 0.98 | 0.85 | 1.13 | 0.819 | 1.02 | 0.90 | 1.15 | 0.755 |
| cg07304943 | 1.03 | 0.85 | 1.24 | 0.772 | 0.897 | 0.98 | 0.80 | 1.20 | 0.833 | 1.01 | 0.89 | 1.15 | 0.849 |
| cg01521674 | 1.05 | 0.88 | 1.25 | 0.586 | 0.897 | 0.98 | 0.83 | 1.16 | 0.841 | 1.01 | 0.89 | 1.14 | 0.866 |
| cg09805314 | 0.93 | 0.68 | 1.27 | 0.634 | 0.897 | 0.95 | 0.51 | 1.80 | 0.886 | 0.94 | 0.71 | 1.24 | 0.650 |
| cg17416748 | 1.03 | 0.88 | 1.20 | 0.731 | 0.897 | 1.01 | 0.85 | 1.20 | 0.905 | 1.01 | 0.90 | 1.13 | 0.885 |
| cg27219662 | 0.97 | 0.77 | 1.21 | 0.779 | 0.897 | 0.99 | 0.82 | 1.20 | 0.937 | 0.99 | 0.85 | 1.14 | 0.861 |
| cg17657594 | 1.04 | 0.89 | 1.22 | 0.583 | 0.897 | 0.98 | 0.67 | 1.44 | 0.938 | 1.06 | 0.92 | 1.21 | 0.436 |
| cg13110966 | 1.08 | 0.82 | 1.42 | 0.580 | 0.897 | 1.00 | 0.74 | 1.34 | 0.976 | 1.04 | 0.85 | 1.26 | 0.725 |
| cg24990422 | 1.08 | 0.57 | 2.07 | 0.810 | 0.897 | 1.00 | 0.70 | 1.43 | 0.990 | 1.03 | 0.76 | 1.40 | 0.829 |
| cg13646645 | 1.06 | 0.63 | 1.78 | 0.824 | 0.899 | 0.77 | 0.47 | 1.27 | 0.307 | 0.89 | 0.63 | 1.26 | 0.497 |
| cg19276059 | 1.01 | 0.91 | 1.12 | 0.839 | 0.904 | 1.06 | 0.94 | 1.20 | 0.336 | 1.04 | 0.96 | 1.12 | 0.363 |
| cg27636813 | 0.98 | 0.81 | 1.20 | 0.869 | 0.927 | 1.01 | 0.82 | 1.24 | 0.934 | 1.00 | 0.87 | 1.15 | 0.980 |
| cg19286631 | 1.01 | 0.89 | 1.14 | 0.884 | 0.933 | 1.03 | 0.99 | 1.07 | 0.146 | 1.02 | 0.99 | 1.06 | 0.162 |
| cg11524778 | 0.98 | 0.72 | 1.34 | 0.909 | 0.949 | 1.09 | 0.77 | 1.54 | 0.617 | 1.05 | 0.83 | 1.32 | 0.679 |
| cg07162905 | 1.02 | 0.75 | 1.37 | 0.919 | 0.949 | 1.02 | 0.77 | 1.37 | 0.875 | 1.00 | 0.81 | 1.23 | 0.985 |
| cg14059339 | 1.00 | 0.99 | 1.02 | 0.932 | 0.952 | 0.99 | 0.98 | 1.01 | 0.242 | 1.00 | 0.99 | 1.01 | 0.386 |
| cg05594872 | 1.00 | 0.96 | 1.04 | 0.960 | 0.970 | 1.01 | 0.98 | 1.04 | 0.596 | 1.00 | 0.98 | 1.03 | 0.809 |
| cg19494464 | 1.00 | 0.70 | 1.43 | 0.988 | 0.988 | 1.17 | 0.84 | 1.62 | 0.350 | 1.05 | 0.83 | 1.33 | 0.676 |

Hazard ratio (HR), 95% confidence interval (95% CI), and *P*-value were derived from Cox proportional hazards model adjusted for age, sex, clinical stage, smoking status, and study centre.

FDR: false discovery rate

**Supplementary** **Table S5.** Results of proportional hazards test for 96 CpG probes of *TRIM27* in LUSC samples

| CPG probe | Discovery phase | | | Validation phase | | Combined dataset | |
| --- | --- | --- | --- | --- | --- | --- | --- |
|  | *χ*^2^ | *P* | FDR-*q* | *χ*^2^ | *P* | *χ*^2^ | *P* |
| cg05293407 | 3.412 | 0.065 | 0.469 | 0.248 | 0.618 | 1.972 | 0.160 |
| cg16578453 | 2.61E-04 | 0.987 | 0.987 |  |  |  |  |
| cg17416748 | 0.001 | 0.982 | 0.987 |  |  |  |  |
| cg27219662 | 0.001 | 0.980 | 0.987 |  |  |  |  |
| cg18578954 | 0.002 | 0.967 | 0.987 |  |  |  |  |
| cg06126060 | 0.004 | 0.952 | 0.987 |  |  |  |  |
| cg20370991 | 0.004 | 0.951 | 0.987 |  |  |  |  |
| cg14781667 | 0.004 | 0.949 | 0.987 |  |  |  |  |
| cg10729419 | 0.005 | 0.944 | 0.987 |  |  |  |  |
| cg14633298 | 0.010 | 0.920 | 0.987 |  |  |  |  |
| cg09444060 | 0.027 | 0.870 | 0.960 |  |  |  |  |
| cg01928896 | 0.033 | 0.856 | 0.955 |  |  |  |  |
| cg21568286 | 0.043 | 0.836 | 0.945 |  |  |  |  |
| cg27383651 | 0.074 | 0.786 | 0.898 |  |  |  |  |
| cg22842085 | 0.097 | 0.755 | 0.874 |  |  |  |  |
| cg11389756 | 0.113 | 0.736 | 0.862 |  |  |  |  |
| cg19519384 | 0.115 | 0.735 | 0.862 |  |  |  |  |
| cg00259404 | 0.127 | 0.721 | 0.862 |  |  |  |  |
| cg23569711 | 0.132 | 0.716 | 0.862 |  |  |  |  |
| cg20986887 | 0.137 | 0.711 | 0.862 |  |  |  |  |
| cg10718056 | 0.137 | 0.711 | 0.862 |  |  |  |  |
| cg17344091 | 0.142 | 0.707 | 0.862 |  |  |  |  |
| cg16723189 | 0.152 | 0.696 | 0.862 |  |  |  |  |
| cg05594872 | 0.208 | 0.648 | 0.841 |  |  |  |  |
| cg13060704 | 0.217 | 0.642 | 0.841 |  |  |  |  |
| cg03270340 | 0.231 | 0.630 | 0.841 |  |  |  |  |
| cg11524778 | 0.254 | 0.614 | 0.830 |  |  |  |  |
| cg15629064 | 0.302 | 0.583 | 0.799 |  |  |  |  |
| cg22900372 | 0.340 | 0.560 | 0.779 |  |  |  |  |
| cg19276059 | 0.352 | 0.553 | 0.779 |  |  |  |  |
| cg16061301 | 0.384 | 0.535 | 0.767 |  |  |  |  |
| cg17564183 | 0.422 | 0.516 | 0.750 |  |  |  |  |
| cg12896170 | 0.444 | 0.505 | 0.746 |  |  |  |  |
| cg05314350 | 0.461 | 0.497 | 0.746 |  |  |  |  |
| cg16754082 | 0.470 | 0.493 | 0.746 |  |  |  |  |
| cg03671443 | 0.477 | 0.490 | 0.746 |  |  |  |  |
| cg08404201 | 0.494 | 0.482 | 0.746 |  |  |  |  |
| cg14059339 | 0.494 | 0.482 | 0.746 |  |  |  |  |
| cg07304943 | 0.514 | 0.473 | 0.746 |  |  |  |  |
| cg07791404 | 0.542 | 0.462 | 0.746 |  |  |  |  |
| cg26723331 | 0.556 | 0.456 | 0.746 |  |  |  |  |
| cg13646645 | 0.560 | 0.454 | 0.746 |  |  |  |  |
| cg17657594 | 0.587 | 0.443 | 0.746 |  |  |  |  |
| cg25589651 | 0.588 | 0.443 | 0.746 |  |  |  |  |
| cg09325158 | 0.619 | 0.432 | 0.746 |  |  |  |  |
| cg05656855 | 0.681 | 0.409 | 0.746 |  |  |  |  |
| cg06600118 | 0.719 | 0.396 | 0.746 |  |  |  |  |
| cg19286631 | 0.769 | 0.380 | 0.730 |  |  |  |  |
| cg23756442 | 0.798 | 0.372 | 0.728 |  |  |  |  |
| cg19400926 | 0.834 | 0.361 | 0.722 |  |  |  |  |
| cg10401017 | 0.930 | 0.335 | 0.684 |  |  |  |  |
| cg19494464 | 0.985 | 0.321 | 0.670 |  |  |  |  |
| cg06395692 | 0.990 | 0.320 | 0.670 |  |  |  |  |
| cg11629443 | 1.002 | 0.317 | 0.670 |  |  |  |  |
| cg17352468 | 1.009 | 0.315 | 0.670 |  |  |  |  |
| cg16959626 | 1.086 | 0.297 | 0.670 |  |  |  |  |
| cg21413754 | 1.101 | 0.294 | 0.670 |  |  |  |  |
| cg08125020 | 1.105 | 0.293 | 0.670 |  |  |  |  |
| cg18954047 | 1.132 | 0.287 | 0.670 |  |  |  |  |
| cg14667731 | 1.213 | 0.271 | 0.670 |  |  |  |  |
| cg27636813 | 1.271 | 0.260 | 0.670 |  |  |  |  |
| cg11362785 | 1.293 | 0.256 | 0.670 |  |  |  |  |
| cg09805314 | 1.299 | 0.254 | 0.670 |  |  |  |  |
| cg14268557 | 1.336 | 0.248 | 0.670 |  |  |  |  |
| cg27120125 | 1.358 | 0.244 | 0.670 |  |  |  |  |
| cg25922680 | 1.406 | 0.236 | 0.670 |  |  |  |  |
| cg00875200 | 1.440 | 0.230 | 0.670 |  |  |  |  |
| cg07162905 | 1.518 | 0.218 | 0.670 |  |  |  |  |
| cg15201399 | 1.582 | 0.208 | 0.670 |  |  |  |  |
| cg24990422 | 1.653 | 0.199 | 0.670 |  |  |  |  |
| cg14700841 | 1.811 | 0.178 | 0.634 |  |  |  |  |
| cg11809702 | 1.879 | 0.170 | 0.629 |  |  |  |  |
| cg13110966 | 1.937 | 0.164 | 0.629 |  |  |  |  |
| cg10905031 | 2.000 | 0.157 | 0.629 |  |  |  |  |
| cg18844029 | 2.309 | 0.129 | 0.537 |  |  |  |  |
| cg20741078 | 2.325 | 0.127 | 0.537 |  |  |  |  |
| cg19284131 | 2.430 | 0.119 | 0.537 |  |  |  |  |
| cg19484381 | 2.466 | 0.116 | 0.537 |  |  |  |  |
| cg25701364 | 2.669 | 0.102 | 0.517 |  |  |  |  |
| cg02593352 | 2.829 | 0.093 | 0.494 |  |  |  |  |
| cg23211158 | 2.991 | 0.084 | 0.473 |  |  |  |  |
| cg13612515 | 3.101 | 0.078 | 0.469 |  |  |  |  |
| cg05216056 | 3.128 | 0.077 | 0.469 |  |  |  |  |
| cg27358207 | 3.188 | 0.074 | 0.469 |  |  |  |  |
| cg03647327 | 3.191 | 0.074 | 0.469 |  |  |  |  |
| cg16520539 | 3.353 | 0.067 | 0.469 |  |  |  |  |
| cg26892251 | 3.700 | 0.054 | 0.469 |  |  |  |  |
| cg18104091 | 3.985 | 0.046 | 0.469 |  |  |  |  |
| cg23780580 | 4.047 | 0.044 | 0.469 |  |  |  |  |
| cg01521674 | 5.012 | 0.025 | 0.345 |  |  |  |  |
| cg11886965 | 5.303 | 0.021 | 0.341 |  |  |  |  |
| cg06021189 | 6.534 | 0.011 | 0.203 |  |  |  |  |
| cg08895903 | 6.552 | 0.010 | 0.203 |  |  |  |  |
| cg18234193 | 7.786 | 0.005 | 0.168 |  |  |  |  |
| cg08356572 | 8.009 | 0.005 | 0.168 |  |  |  |  |
| cg15613012 | 10.927 | 0.001 | 0.091 |  |  |  |  |

**Supplementary Table S6.** Comparison of smoking-related characteristics of former and current smokers between early-stage LUAD and LUSC

| Variable | LUAD  (*N* = 824) | LUSC  (*N* = 406) | Statistic | *P* |
| --- | --- | --- | --- | --- |
| Smoking |  |  |  |  |
| No (never/former smokers) | 582 (71.76%) | 261 (65.91%) | *χ*^2^ = 4.33 | 0.037 |
| Yes (current smokers) | 229 (28.24%) | 135 (34.09%) |  |  |
| Unknown | 13 | 10 |  |  |
| Pack-year of smoking^a^ | 37.49 ± 29.39 | 54.79 ± 31.23 | *z* = 72458 | 1.03×10^-19^ |
| Years of smoking^b^ | 36.62 ± 16.86 | 44.13 ± 12.57 | *z* = 38812 | 1.83×10^-8^ |
| Years of smoking cessation^c^ | 11.37 ± 13.18 | 8.96 ± 10.16 | *z* = 40912 | 0.597 |

^a^ A pack-year was defined as twenty cigarettes smoked every day for one year.

^b^ Years of smoking indicated the number of years the patient has smoked.

^c^ Year of smoking cessation indicated the number of years the patient quit smoking and was only analyzed in former smokers.

LUAD: lung adenocarcinoma; LUSC: lung squamous cell carcinoma

**Supplementary Table S7.** Results of genome-wide methylation transcription analysis of 29 genes significantly associated with cg05293407 in TCGA LUSC samples

| Gene | Coefficient | 95% CI | | Standard error | *t* | *P* | FDR-*q* |
| --- | --- | --- | --- | --- | --- | --- | --- |
| *BAT3* | 17.97 | 10.42 | 25.52 | 3.84 | 4.68 | 4.22E-06 | 0.01 |
| *C10orf82* | 76.78 | 42.12 | 111.44 | 17.62 | 4.36 | 1.77E-05 | 0.03 |
| *C19orf41* | 37.47 | 23.93 | 51.01 | 6.88 | 5.44 | 1.05E-07 | 7.15E-04 |
| *CCL4L2* | -54.74 | -80.60 | -28.88 | 13.14 | -4.16 | 4.03E-05 | 0.04 |
| *CDH19* | 76.79 | 39.39 | 114.20 | 19.01 | 4.04 | 6.74E-05 | 0.05 |
| *CLEC4F* | 53.65 | 28.06 | 79.24 | 13.01 | 4.13 | 4.74E-05 | 0.04 |
| *CMTM5* | 49.78 | 27.81 | 71.75 | 11.17 | 4.46 | 1.15E-05 | 0.02 |
| *CXorf48* | 68.37 | 37.51 | 99.23 | 15.68 | 4.36 | 1.77E-05 | 0.03 |
| *DIS3L2* | 18.53 | 10.44 | 26.63 | 4.11 | 4.51 | 9.31E-06 | 0.02 |
| *DUXA* | 30.00 | 17.37 | 42.64 | 6.42 | 4.67 | 4.39E-06 | 0.01 |
| *GAS2* | 63.53 | 33.31 | 93.75 | 15.36 | 4.14 | 4.52E-05 | 0.04 |
| *GBX1* | 25.23 | 13.67 | 36.79 | 5.87 | 4.29 | 2.33E-05 | 0.03 |
| *GJC3* | 46.06 | 27.31 | 64.81 | 9.53 | 4.83 | 2.09E-06 | 0.01 |
| *HACE1* | 33.74 | 21.63 | 45.86 | 6.16 | 5.48 | 8.64E-08 | 7.15E-04 |
| *KIAA0776* | 16.27 | 8.50 | 24.04 | 3.95 | 4.12 | 4.87E-05 | 0.04 |
| *NAALAD2* | 59.50 | 35.04 | 83.97 | 12.43 | 4.79 | 2.62E-06 | 0.01 |
| *NKX6.2* | 17.49 | 9.05 | 25.94 | 4.29 | 4.07 | 5.83E-05 | 0.04 |
| *OR56A3* | 38.18 | 20.50 | 55.87 | 8.99 | 4.25 | 2.83E-05 | 0.03 |
| *RNF5* | 19.76 | 10.34 | 29.19 | 4.79 | 4.12 | 4.74E-05 | 0.04 |
| *SIRPG* | -60.90 | -87.84 | -33.96 | 13.69 | -4.45 | 1.20E-05 | 0.02 |
| *SUGT1P1* | 42.96 | 22.48 | 63.44 | 10.41 | 4.13 | 4.71E-05 | 0.04 |
| *TBX22* | 41.03 | 28.62 | 53.45 | 6.31 | 6.50 | 3.05E-10 | 6.26E-06 |
| *TCEAL5* | 46.35 | 24.09 | 68.61 | 11.32 | 4.10 | 5.34E-05 | 0.04 |
| *TRIM39* | 18.83 | 10.03 | 27.64 | 4.48 | 4.21 | 3.36E-05 | 0.03 |
| *UGT3A1* | 89.92 | 48.07 | 131.76 | 21.27 | 4.23 | 3.09E-05 | 0.03 |
| *USP26* | 26.88 | 14.35 | 39.41 | 6.37 | 4.22 | 3.20E-05 | 0.03 |
| *ZNF184* | 21.88 | 11.98 | 31.78 | 5.03 | 4.35 | 1.86E-05 | 0.03 |
| *ZNF187* | 22.87 | 13.26 | 32.49 | 4.89 | 4.68 | 4.26E-06 | 0.01 |
| *ZSCAN12* | 32.98 | 18.99 | 46.96 | 7.11 | 4.64 | 5.14E-06 | 0.01 |

Linear regression model adjusted for age, sex, clinical stage, and smoking status.

TCGA: The Cancer Genome Atlas; LUSC: lung squamous cell carcinoma; CI: confidence interval; FDR: false discovery rate
